# Hsp70 and DNAJA2 limit CFTR levels through degradation

**DOI:** 10.1371/journal.pone.0220984

**Published:** 2019-08-13

**Authors:** Patrick Kim Chiaw, Christine Hantouche, Michael J. H. Wong, Elizabeth Matthes, Renaud Robert, John W. Hanrahan, Alvin Shrier, Jason C. Young

**Affiliations:** 1 Department of Biochemistry, McGill University, Montreal, Quebec, Canada; 2 Groupe de Recherche Axé sur la Structure des Protéines, McGill University, Montreal, Quebec, Canada; 3 Department of Physiology, McGill University, Montreal, Quebec, Canada; University of Pittsburgh, UNITED STATES

## Abstract

Cystic Fibrosis is caused by mutations in the CFTR anion channel, many of which cause its misfolding and degradation. CFTR folding depends on the Hsc70 and Hsp70 chaperones and their co-chaperone DNAJA1, but Hsc70/Hsp70 is also involved in CFTR degradation. Here, we address how these opposing functions are balanced. DNAJA2 and DNAJA1 were both important for CFTR folding, however overexpressing DNAJA2 but not DNAJA1 enhanced CFTR degradation at the endoplasmic reticulum by Hsc70/Hsp70 and the E3 ubiquitin ligase CHIP. Excess Hsp70 also promoted CFTR degradation, but this occurred through the lysosomal pathway and required CHIP but not complex formation with HOP and Hsp90. Notably, the Hsp70 inhibitor MKT077 enhanced levels of mature CFTR and the most common disease variant ΔF508-CFTR, by slowing turnover and allowing delayed maturation, respectively. MKT077 also boosted the channel activity of ΔF508-CFTR when combined with the corrector compound VX809. Thus, the Hsp70 system is the major determinant of CFTR degradation, and its modulation can partially relieve the misfolding phenotype.

## Introduction

Cystic Fibrosis (CF) is an autosomal recessive genetic disease which results from mutations in the gene encoding the Cystic Fibrosis transmembrane conductance regulator (CFTR, gene *ABCC7*). CFTR normally resides at the apical surface of epithelial cells where it mediates the flux of chloride and bicarbonate ions across the membrane [1, 2]. The most prevalent disease mutation is the deletion of phenylalanine 508 (ΔF508-CFTR), which leads to misfolding and retention of the protein in the endoplasmic reticulum (ER) where it is targeted for degradation through the ER associated degradation (ERAD) pathway [3]. Therefore elucidating the molecular mechanisms of CFTR folding and degradation remains a high priority for understanding CF pathogenesis and development of new therapeutics.

CFTR is a member of the ATP-binding cassette (ABC) transporter superfamily which contains 1480 amino acid residues and forms five subdomains; two membrane-spanning domains (MSD1 and MSD2) each with six transmembrane domains, two nucleotide-binding domains (NBD1 and NBD2), and a regulatory (R) domain [4]. CFTR synthesis at the ER requires approximately 10 minutes and requires co- and post-translational folding events that involve the cooperative assembly of N- and C-terminal membrane and cytosolic subdomains [5–8]. CFTR assembly progresses through the formation of different folding intermediates, however CFTR folding is relatively inefficient and much of the newly-synthesized channel is degraded [3]. The F508 residue is located on the surface of NBD1 and is important for folding and proper inter-domain assembly with NBD2 and MSD2, and thus the trafficking of CFTR to the plasma membrane (PM) [6, 7, 9]. Consequently, the great majority of ΔF508-CFTR is targeted for ERAD.

Molecular chaperones assist the folding and assembly of the cytosolic domains of CFTR. Hsc70 (*HSPA8*), its inducible homolog Hsp70 (*HSPA1A/B*), and Hsp90 are thought to be the most important [10–12]. Hsc70 and Hsp70 are activated by the J domains of DNAJ co-chaperones, to support folding but also a variety of other processes [13, 14]. Hsc70 and the co-chaperone DNAJA1 (also called DJA1/Hdj2) assist co-translational folding of NBD1 at the ER [15]. Using a proteomic approach, it was found that more Hsc70/Hsp70 was associated with misfolded ΔF508-CFTR than wild-type (WT) [16]. The Hsp90 system is involved most probably in the later steps of CFTR folding, as Hsp90 inhibition blocks CFTR maturation and accelerates its degradation [10]. In addition, downregulation of the Hsp90 co-chaperone Aha1 partially rescues ΔF508-CFTR allowing its accumulation at the cell surface [11].

Molecular chaperones are also involved in the degradation of proteins, including CFTR. The cytosolic E3 ubiquitin ligase CHIP interacts with Hsc70/Hsp70 to promote poly-ubiquitination and ERAD of misfolded CFTR [17–19]. Thus, Hsc70/Hsp70 aids the opposing processes of CFTR folding and degradation. In addition, there is an ER membrane-associated complex that involves the E3 ligases RMA1 and gp78 which cooperate to poly-ubiquitinate CFTR [20, 21]. Poly-ubiquitinated CFTR is sent for proteasomal degradation through a pathway that involves derlin-1 [20], BAP31 [22], and p97 [23]. RMA1 has been proposed to act co-translationally to sense the folding status and assembly of NBD1 and the R domain while Hsc70-CHIP may act post-translationally after NBD2 synthesis to detect folding defects that involve terminal steps in CFTR assembly [20].

Various DNAJs have different effects on the ER folding or degradation of CFTR. DJA1 promotes poly-ubiquitination of NBD1 by Hsc70 and CHIP in reconstitution experiments [24]. However, DJA1 knockdown decreases CFTR folding and trafficking, suggesting that its chaperone role outweighs its ERAD role [25]. DNAJB1 (DJB1/Hdj1/Hsp40), a major stress-induced co-chaperone, stabilizes immature CFTR but has no overall effect on trafficking [26]. CSPα (DNAJC5) associates with the ER membrane, and promotes CFTR ERAD through formation of complexes with Hsc70 and CHIP [27]. However, CSPα binds CHIP independently of Hsc70, in contrast to DJA1 which interacts directly with NBD1 [15, 27]. Transmembrane ER resident DNAJB12 promotes degradation of misfolded CFTR through Hsc70 and RMA1, instead of CHIP [25, 28]. DNAJA2 (DJA2) is highly conserved with DJA1, but is less well studied. We found that it is biologically distinct from DJA1: only DJA2 could promote folding of the model substrate luciferase, but both promoted CHIP-mediated degradation of the hERG potassium channel at the ER [29, 30]. The role of DJA2 with regard to CFTR at the ER remains unclear.

The Hsc70-CHIP complex also functions in cell surface quality control by promoting lysosomal degradation of mature misfolded CFTR at the plasma membrane (PM). Hsp90 is also implicated in this function, as well as the co-chaperone HOP [31], which connects Hsp90 with Hsc70 [32, 33]. Interestingly, while Hsc70 and DJA1 act in mature CFTR degradation, Hsp70 and DJA2 have little effect [31]. In contrast, DJA2 and Hsc70 maintain the channel activity of misfolded mature ΔF508-CFTR, whereas DJA1 and Hsp70 cannot [34]. Artificial induction of a heat shock response, which includes upregulation of Hsp70, Hsp90 and DJB1, also promotes ΔF508-CFTR activity at the PM, but a similar effect was achieved by small molecule activation of Hsp90 alone [34]. A question then arises about the role of Hsp70 at the PM, and by extension at the ER, as most early experiments focused on Hsc70.

In recent years, small molecule inhibitors of Hsc70/Hsp70 have become available, and many are being studied for their anti-tumour properties. Apoptozole and VER155008 fill the ATP-binding pocket of Hsc70/Hsp70 and compete with nucleotide binding [35–38]. 2-Phenylethynesulfonamide/pifithrin-μ and related compounds target the substrate binding domain [39, 40]. Other compounds, including MKT077 and YK5 and their derivatives, act allosterically on the nucleotide-binding domain without competing for nucleotide [41–45]. These compounds are important new tools for assessing the role of Hsp70 chaperones in cells.

There has in addition been much effort in finding small molecules that improve the trafficking or channel function of CFTR mutants directly [46]. Corrector compounds such as VX809 stabilize the structure of ΔF508-CFTR [47–49]. VX809 (lumacaftor) is approved for clinical use in combination with another compound VX770 (ivacaftor) that enhances channel activity [50, 51]. Although this treatment is only partially effective, drug combinations are expected to be the future of CF pharmacotherapy [52, 53].

While the folding and degradation roles of Hsc70/Hsp70 have been studied separately, whether the net activity favours maturation or ERAD of CFTR has not been directly addressed. Therefore, we examined the net effects of the Hsc70/Hsp70 chaperones and the DJA1 and DJA2 co-chaperones for CFTR, using knockdown and overexpression experiments. Remarkably, Hsp70 negatively affected mature CFTR amounts. We identified effects of DJA2 on ERAD, and Hsp70 on lysosomal degradation of CFTR. Furthermore, MKT077 rescued levels of mature wild-type and ΔF508-CFTR, enhancing channel activity when combined with VX809. We propose a model in which a balance of chaperone activities regulate the degradation of CFTR in cells.

## Results

### Optimum levels of Hsp70 and DJAs are required for mature CFTR

**Fig 1:**
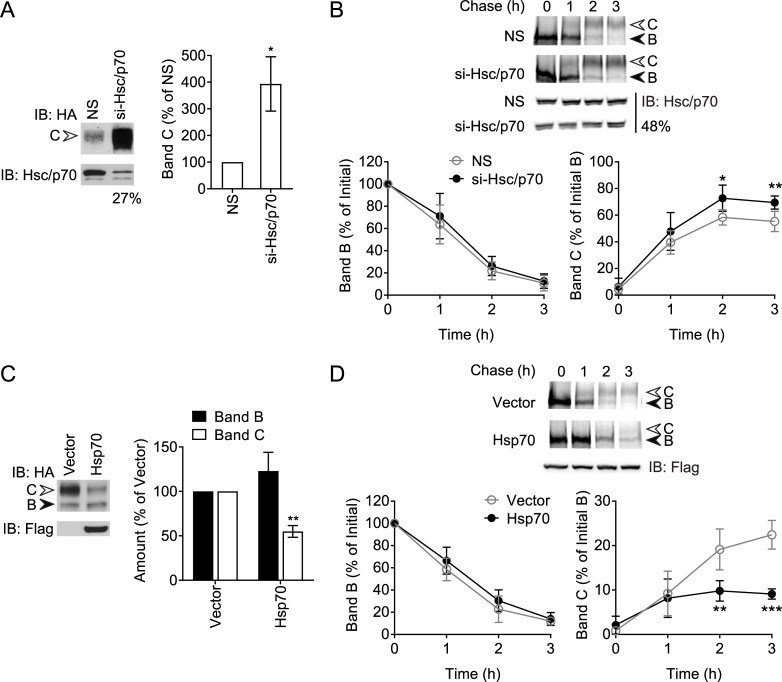
Hsc70/Hsp70 suppresses levels of mature CFTR. (A) Immunoblot (IB) of CFTR-3HA stably expressed in HeLa cells and transfected with siRNA against Hsc70 and Hsp70 (si-Hsc/p70) or non-silencing (NS) siRNA. Mature complex-glycosylated band C and immature core-glycosylated band B forms of CFTR are marked. Knockdown of Hsc70/Hsp70 was monitored by immunoblot and quantified as percentage of non-silencing control. Quantitation of band C is shown relative to amounts in non-silencing control, n = 3. (B) Pulse-chase autoradiograph of CFTR-3HA in HeLa cells treated as in (A). Knockdown of Hsc70/Hsp70 was monitored by immunoblot and quantified as percentage of non-silencing control. Quantitations of bands B and C are shown relative to initial amounts of band B, n = 5. (C) Immunoblot of HEK293 cells transfected with CFTR-3HA and Flag-Hsp70 or vector control. Expression of Flag-Hsp70 was detected by immunoblot. Quantitation of bands B and C are shown relative to vector control, n = 3. (D) Pulse-chase autoradiograph of CFTR-3HA in HEK293 cells treated as in (C). Expression of Flag-Hsp70 was monitored by immunoblot. Quantitations of bands B and C are shown, n = 6. Error bars show standard deviation from the mean, * p<0.05, ** p<0.01, *** p<0.001.

Hsc70/Hsp70 has opposing roles in CFTR biosynthetic folding and ERAD. To determine which role may be predominant, they were depleted by knockdown using a mixture of three siRNA duplexes, in HeLa cells stably expressing 3HA-tagged CFTR. Total Hsc70 and Hsp70 expression was depleted to around 27% of non-silencing control (Fig 1A). Mature CFTR, detected at steady-state as the complex-glycosylated band C form (~170 kDa), remarkably increased around 4-fold in amount upon Hsc70/Hsp70 knockdown (Fig 1A). To address effects on CFTR biosynthesis, maturation kinetics were measured using radiolabeled pulse-chase experiments upon knockdown of Hsc70/Hsp70 to around 48% of control. CFTR was radiolabelled in the above cells and maturation was monitored over a 3 h chase. Consistent with previous reports [3, 31, 54], immature core-glycosylated band B CFTR at the ER (~140 kDa) disappeared rapidly over the course of 3 h, with a fraction of immature CFTR trafficking past the Golgi to be processed into mature band C (Fig 1B). Although the rate of band B disappearance was not affected by Hsc70/Hsp70 depletion, band C was significantly increased above the control (Fig 1B). The larger effect observed at steady-state (Fig 1A) suggested that the small biosynthetic effect may be cumulative, or that PM quality control is also being affected. Although mature CFTR is known to be degraded in lysosomes [31], addition of the lysosome inhibitor chloroquine had no effect on the pulse-chase, confirming that only biosynthetic trafficking was being measured (S1 Fig).

Depletion of Hsc70/Hsp70 led to a partial heat shock response (HSR) with increases in DJB1, Hsp90, and to a small degree DJA1 (S2A Fig). DJB1 alone does not affect CFTR trafficking, but the increase in Hsp90 may contribute to the effects in Fig 1A and 1B. However, activation of Hsp90 is thought to be limiting for its function, rather than its total amount, as observed with co-chaperones and small molecules [11, 34]. A modest unfolded protein response (UPR) was observed by upregulation of BiP but not CHOP, compared to the thapsigargin positive control (S2A and S2B Fig). However, the UPR decreases trafficking and increases ERAD of CFTR [55], opposite to our observations. The depletion of Hsc70/Hsp70 is thus likely to have some direct effect on CFTR.

The reciprocal experiments with Hsp70 overexpression were conducted. Hsp70 was used because its expression in HEK293 cells was much higher than that of Hsc70 when co-transfected with CFTR, and at a level comparable to total endogenous Hsc70 and Hsp70 [44]. At steady state, CFTR band C was decreased to about 50% with Hsp70 compared to vector control (Fig 1C). In pulse-chase experiments, approximately 20% of initially radiolabelled band B matured into band C (Fig 1D), consistent with previous studies [3, 5]. Hsp70 overexpression reduced the amount of mature band C present by around half compared to vector control, although it did not alter the disappearance of band B (Fig 1D). The knockdown and overexpression experiments thus yielded internally consistent results that suggest Hsc70/Hsp70 suppresses the level of mature CFTR.

**Fig 2:**
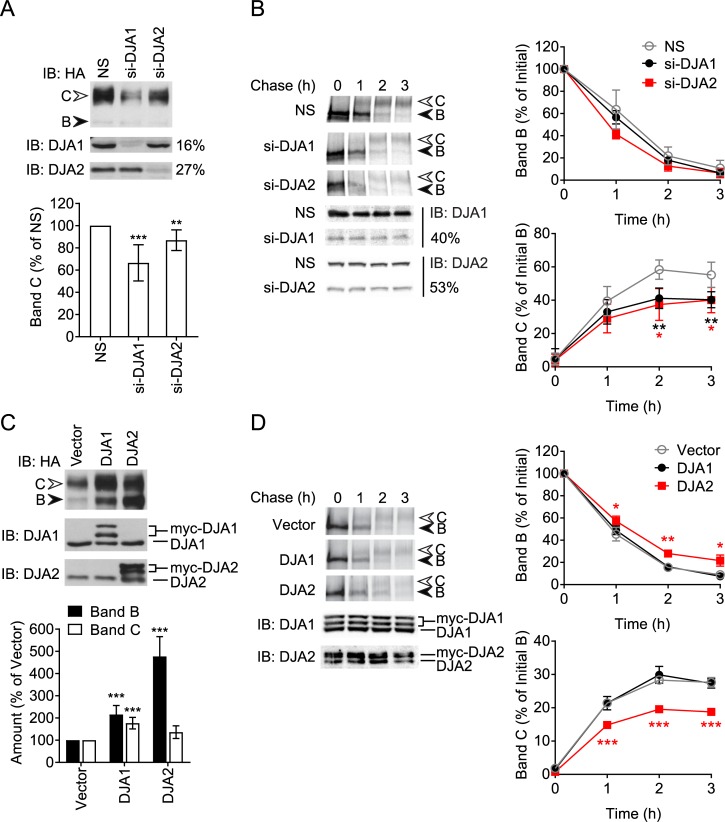
Optimal levels of DJA2 are important for mature CFTR. (A) Immunoblot (IB) of CFTR-3HA stably expressed in HeLa cells and transfected with siRNA against DJA1 (si-DJA1) or DJA2 (si-DJA2) or non-silencing (NS) siRNA. Knockdown of DJA1 and DJA2 was monitored by immunoblot and quantified as percentage of non-silencing control. Quantitation of band C is shown relative to amounts in non-silencing control, n = 8. (B) Pulse-chase autoradiograph of CFTR-3HA in HeLa cells treated as in (A). Knockdown of DJA1 and DJA2 was monitored by immunoblot and quantified as percentage of non-silencing control. Quantitations of bands B and C are shown relative to initial amounts of band B, n = 4. (C) Immunoblot of HEK293 cells transfected with CFTR-3HA and myc-DJA1, myc-DJA2 or vector control. Expression of myc-DJA1 and myc-DJA2 was detected by immunoblot, appearing as double bands due to variable processing [30]. Quantitation of bands B and C are shown relative to vector control, n = 8. (D) Pulse-chase autoradiograph of CFTR-3HA in HEK293 cell treated as in (C). Expression of myc-DJA1 and myc-DJA2 was monitored by immunoblot. Quantitations of bands B and C are shown, n = 3. Error bars show standard deviation from the mean, * p<0.05, ** p<0.01, *** p<0.001.

We further investigated the co-chaperones DJA1 and DJA2. By assisting either folding or CHIP-mediated degradation, they may also have opposing roles. At steady-state, knockdown of DJA1 decreased band C, and depletion of DJA2 had a modest but still significant effect (Fig 2A). In pulse-chase assays, knockdown of DJA1 and DJA2 caused identical decreases in the mature band C produced relative to non-silencing conditions, while band B disappearance was not affected (Fig 2B). Co-chaperone depletion showed little sign of HSR or UPR (S2A and S2B Fig). These results suggest that DJA2 may also have a role in CFTR folding and trafficking.

The effects of overexpressing DJA1 or DJA2 were then compared. At steady state, DJA1 increased the total amount of bands B and C to similar degrees (Fig 2C). Unexpectedly, DJA2 changed the distribution between bands B and C, so that the immature form predominated (Fig 2C). Band B shifted from around 30% of total CFTR in the vector control or with DJA1, to around 60% with DJA2. The large increase in total CFTR with DJA2 was most likely due to a large change in CFTR mRNA, around 3-fold above vector control, caused by DJA2 co-expression, while DJA1 had no such effect (S3 Fig). Taking expression level into account, DJA2 may have an overall negative effect on CFTR trafficking. In pulse-chase experiments, expression differences are accounted for by normalizing to the pulse label before chase and it was observed that DJA2 caused a decrease in band C levels below that of vector control conditions, and a small increase in band B (Fig 2D). In contrast, DJA1 overexpression had no effect on CFTR trafficking kinetics.

These data suggest that although DJA1 can promote both folding and degradation by Hsc70 and CHIP, its net effect in cells favours CFTR maturation. In contrast, the net effect of Hsc70/Hsp70 is to inhibit trafficking. DJA2 may be less important for biosynthetic folding of CFTR, but in excess it interferes with maturation. We propose that an optimum balance of Hsc70/Hsp70 and its DJA co-chaperones is needed for CFTR maturation, and that CFTR is highly sensitive to disruption of the chaperone system.

### DJA2 and Hsp70 promote degradation of CFTR

**Fig 3:**
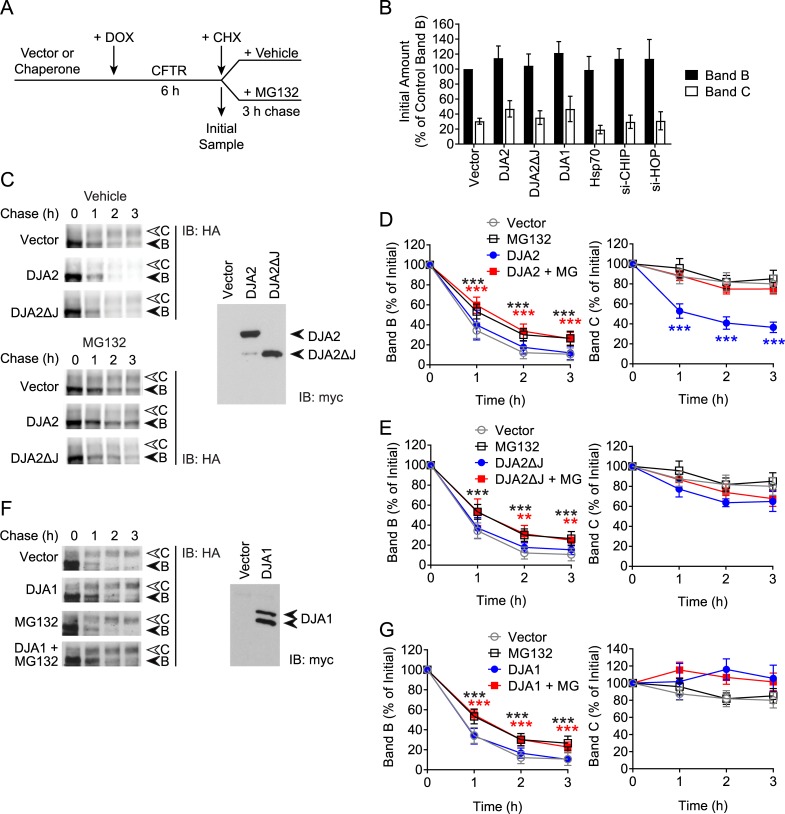
DJA2 decreases maturation of CFTR. (A) Schematic of CFTR induction and cycloheximide chase experiments. (B) Quantitation of CFTR-3HA expression in HEK293 Tet-On cells detected by immunoblot after 6 h doxycycline treatment with different co-expression conditions. The amount of band B in the vector control is set to 100% for comparisons of both band B and C levels. (C) Immunoblot of CFTR-3HA induced for 6 h, then chased with CHX for 0 to 3 h, in the presence of co-expressed myc-DJA2, myc-DJA2ΔJ or vector control. Parallel experiments contained MG132 or vehicle control. Expression of myc-DJA2 and myc-DJA2ΔJ was monitored by immunoblot. (D) Quantitations of CFTR bands B and C in the presence of DJA2 from (C) are shown relative to initial amounts at the start of CHX chase, n = 8 with vehicle control, n = 6 with MG132. (E) Quantitations of CFTR bands B and C in the presence of DJA2ΔJ from (C) are shown relative to initial amounts, n = 6. (F) Immunoblot of CFTR-3HA induced and chased as in (C) except with co-expressed myc-DJA1. (G) Quantitations of CFTR bands B and C in the presence of DJA1 from (F) are shown relative to initial amounts, n = 4. Error bars show standard deviation from the mean, * p<0.05, ** p<0.01, *** p<0.001.

The effects of DJA depletion could be attributed to impaired folding of CFTR, but the loss of mature CFTR caused by overexpression of DJA2 could be more complex. One possibility is that ERAD of newly synthesized CFTR could be increased. Alternatively, overexpression of DJA2 may delay export of CFTR from the ER as evidenced by a moderate increase in immature band B CFTR (Fig 2D). To address these questions efficiently, we used HEK293-Tet-On cells transfected with doxycycline-inducible CFTR, with DJA2 co-expressed under a constitutive CMV promoter (Fig 3A). This allowed us to turn on CFTR expression in an environment with a defined pre-existing population of DJA2 for comparison to vector control conditions. This approach has the added advantage that both immature band B and mature band C are observed in a single experiment. After 6 h of induction, bands B and C were both visible, and the amount of band C produced represented approximately 30% of the band B levels (Fig 3B and 3C), consistent with the pulse-chase studies (Fig 2D). Neither overexpression nor knockdown of chaperones or co-chaperones used below, affected starting levels of bands B and C after 6 h induction (Fig 3B).

Following the 6 h induction, cells were chased in cycloheximide (CHX) for 3 h under different conditions (Fig 3A). In control conditions, less than 20% of initial band B remained, whereas 80% of band C remained after 3 h (Fig 3C and 3D). The kinetics of band B disappearance was similar to that observed by pulse-chase and representing normal biosynthesis (Fig 2D). Consistent with the pulse-chase results, DJA2 overexpression caused a steep decrease in band C to below 40% of the initial levels, compared to 80% in the vector control, with no difference observed in band B levels (Fig 3C and 3D). To verify that the reduction in CFTR band C levels was specific to functional DJA2, we tested the effect with a deletion mutant lacking the J-domain (DJA2ΔJ), shown to be defective in interaction with Hsp70 [30]. Overexpression of DJA2ΔJ did not alter band B levels, and contrary to DJA2 overexpression, only mildly decreased band C levels relative to vector control (Fig 3C and 3E).

To address if DJA2 accelerates ERAD of CFTR, we performed experiments in the presence of the proteasome inhibitor MG132. In control cells, MG132 significantly increased the amount of band B, however this did not translate into an increased band C level (Fig 3C and 3D**)**. When DJA2 was overexpressed, MG132 treatment increased band B levels and restored band C levels to those originally observed in control cells (Fig 3C and 3D). This suggests that DJA2 increases ERAD of band B, resulting in less CFTR being available to mature into band C. MG132 treatment of cells overexpressing DJA2ΔJ also mimicked vector control conditions (Fig 3C and 3E). As expected, DJA1 overexpression had no effect on band B or C kinetics (Fig 3F and 3G). These results demonstrate that overexpression of DJA2 specifically targets CFTR for degradation through an ERAD pathway.

**Fig 4:**
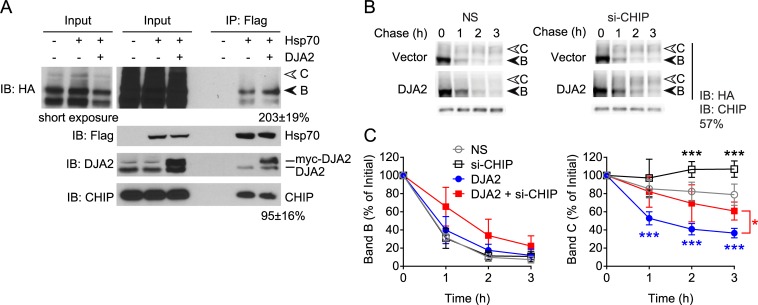
DJA2 promotes CFTR degradation via CHIP. (A) CFTR-3HA was induced for 6 h as in Fig 3 except in the presence of MG132, with co-expressed Flag-Hsp70, or Flag-Hsp70 and myc-DJA2, or vector control. Flag-Hsp70 was immunoprecipitated (IP) and bound CFTR and CHIP detected by immunoblot (IB). Quantified CFTR and CHIP was normalized to the amount of Hsp70 in the IP, and shown as a percentage of the IP with Flag-Hsp70 and without myc-DJA2, n = 3. The species below CFTR band B is an antibody cross reaction that may be a non-glycosylated degradation intermediate. (B) Immunoblot of CFTR-3HA induced and chased as in Fig 3 with co-expressed myc-DJA2 or vector control, with siRNA against CHIP or non-silencing (NS) siRNA. CHIP knockdown was monitored by immunoblot and quantified. (C) Quantitations of CFTR bands B and C from (B) are shown relative to initial amounts, n = 4. Error bars show standard deviation from the mean, * p<0.05, *** p<0.001.

A possible mechanism by which DJA2 accelerates CFTR degradation is by direct binding of Hsc70/Hsp70 complexes with CHIP to CFTR. This would be consistent with the requirement for the J domain shown above, to activate polypeptide binding by Hsc70/Hsp70. However, DNAJB12 promotes ERAD independently of CHIP [25], so other mechanisms were possible. We thus asked whether DJA2 increases the amount of CFTR bound by Hsp70. CFTR was induced for 6 h, in the presence of overexpressed Hsp70, and either DJA2 or vector control. To prevent differences in degradation due to DJA2, the induction was carried out in the presence of MG132. The FLAG-tagged Hsp70 was immunoprecipitated, and bound CFTR detected. DJA2 overexpression caused a 2.1-fold increase in CFTR bound by Hsp70 (Fig 4A). The amount of CHIP bound by Hsp70 remained constant (Fig 4A). These observations, together with the DJA2ΔJ results in Fig 3, suggest that DJA2 acts by recruiting CFTR to Hsp70-CHIP complexes, without directly affecting CHIP itself.

We next examined the induction-chase kinetics of CFTR upon CHIP knockdown. Endogenous CHIP expression levels were depleted to approximately 57% of non-silencing controls (Fig 4B). In control conditions without DJA2 transfection, CHIP depletion moderately increased band C to around 105% of initial levels, compared to the 80% in non-silencing conditions (Fig 4B and 4C). Although no change in band B levels was observed, a decrease in CHIP-mediated ERAD appeared to allow increased maturation. CHIP depletion in DJA2 overexpressed conditions was able to restore band C above the levels in non-silencing controls overexpressing DJA2 (Fig 4B and 4C). These results are consistent with the MG132 studies above and support the conclusion that excess DJA2 specifically promotes ERAD of CFTR through Hsc70/Hsp70 and CHIP, leading to impaired forward trafficking.

**Fig 5:**
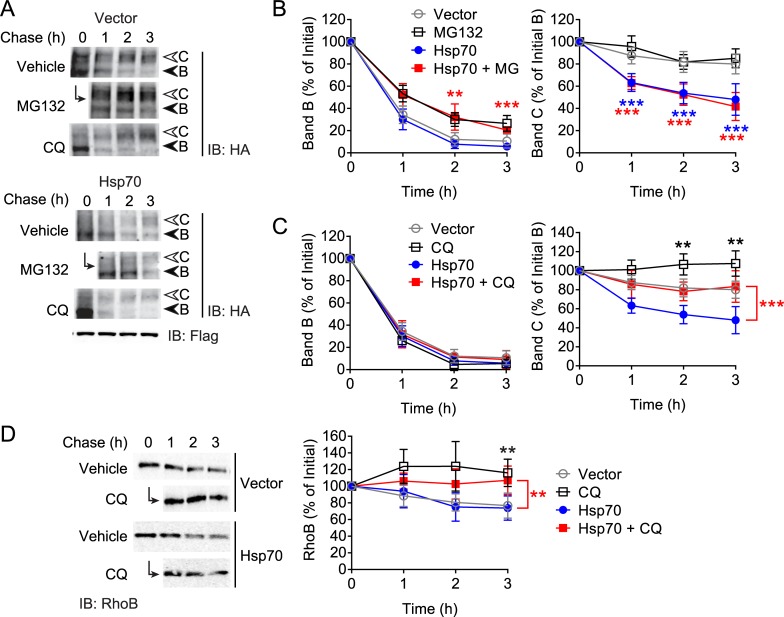
Hsp70 promotes lysosomal degradation of CFTR. (A) Immunoblot of CFTR-3HA induced and chased as in Fig 3 with co-expressed Flag-Hsp70 or vector control, in the presence of either MG132, CQ, or vehicle control. (B) Quantitations of CFTR bands B and C from (A) are shown relative to initial amounts, without or with MG132 treatment, n = 5. Control data for MG132 treatment from Fig 3D are shown here for comparison. (C) Quantitations of CFTR bands B and C from (A) are shown relative to initial amounts, without or with CQ treatment, n = 4. (D) Immunoblot of 3 h CHX chase of HEK293 Tet-On cells transfected with RhoB and either Flag-Hsp70 or vector control, without or with CQ treatment. Quantitation of RhoB is shown relative to initial amounts at the start of chase, n = 5. Error bars show standard deviation from the mean, * p<0.05, ** p<0.01, *** p<0.001.

We next investigated effects of Hsp70 overexpression on CFTR using the induction-chase protocol. Similar to the pulse-chase experiments (Fig 1D), overexpression of Hsp70 led to a rapid decline in band C compared to vector control, to around 45% of initial levels (Fig 5A and 5B). As we established that DJA2 promoted ERAD of immature CFTR through CHIP, it seemed likely that overexpressed Hsp70 acted the same way. MG132 treatment of cells overexpressing Hsp70 displayed increased band B levels in a similar fashion to vector control conditions, but had no effect on restoring the loss of band C (Fig 5A and 5B). These results are contrary to those with DJA2 (Fig 3C and 3D) suggesting that Hsp70 suppresses mature CFTR through mechanisms other than ERAD.

As Hsp70 overexpression did not change the levels of band B during the chase, there was no indication that forward trafficking of CFTR was impaired. Therefore we addressed whether Hsp70 overexpression directed mature CFTR towards degradation. Induction-chase experiments were performed in the presence of chloroquine (CQ) to inhibit lysosomal proteases by raising the organellar pH. In vector transfected cells, and as expected, CQ did not affect band B, but caused a moderate increase in band C during the chase (Fig 5A and 5C). However, CQ treatment of cells overexpressing Hsp70 was able to restore band C levels to that in the control cells (Fig 5A and 5C). Taken together, our results suggest that excess Hsp70 acts primarily to target mature CFTR for lysosomal degradation, while DJA2, when in excess, works with endogenous levels of Hsc70/Hsp70 to favour ERAD of CFTR.

To test whether Hsp70 overexpression accelerates lysosomal degradation of substrates in a non-specific manner, we assessed the degradation of the bona fide lysosome substrate RhoB [56]. In vector-transfected conditions, 20% of RhoB is degraded within 3 h of CHX chase, but CQ treatment prevented turnover (Fig 5D). Overexpression of Hsp70 did not alter the starting levels of RhoB, nor the rate of degradation (Fig 5D), thus suggesting that Hsp70 specifically directs CFTR for lysosomal degradation.

Previous studies from Lukacs *et al*. identified a role for Hsc70/Hsp70 and its co-chaperones in internalization and lysosomal targeting of rescued ΔF508-CFTR from the plasma membrane [31]. Using siRNA screening, they showed that Hsc70 but not Hsp70 was required, and that CHIP ubiquitination was the major mechanism. Furthermore, there was evidence of roles for DJA1 and the co-chaperone HOP that links Hsc70/Hsp70 to Hsp90 [31, 33]. Here, our results on native, wild-type CFTR suggest that Hsp70 is important for its lysosomal targeting, and there was no evidence of a role for DJA1. Hsc70 co-transfection levels in HEK293 were too low for us to make reliable conclusions, thus, the involvement of CHIP and HOP in Hsp70 directed lysosomal degradation was examined.

**Fig 6:**
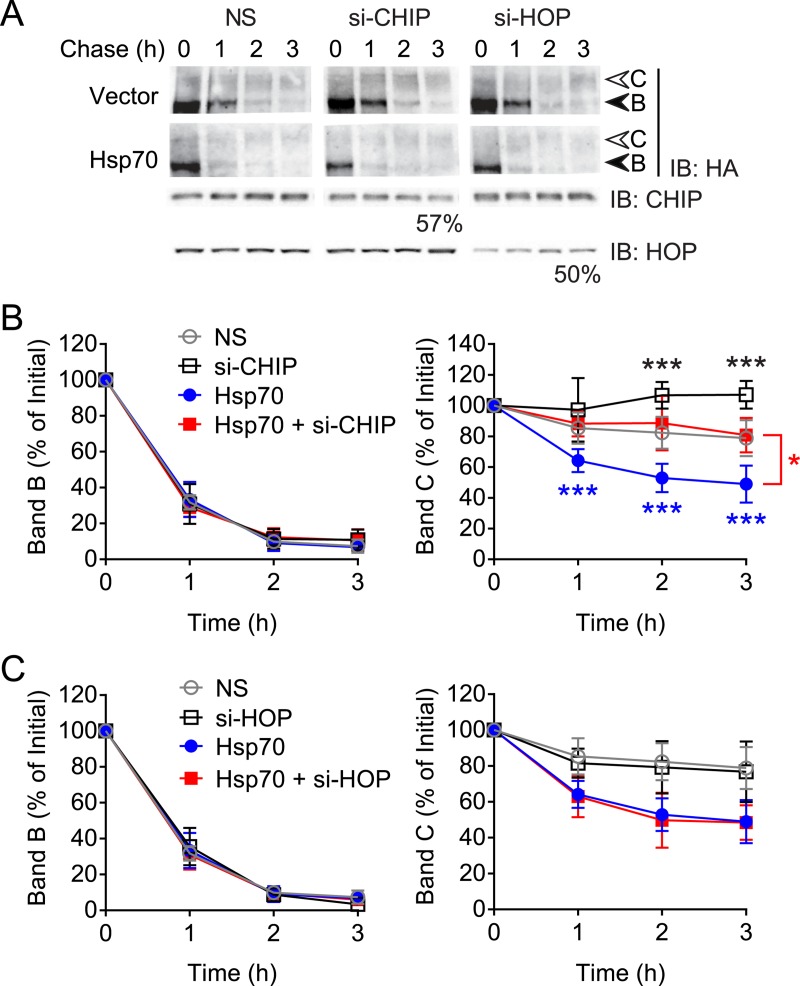
CHIP but not HOP acts in Hsp70-mediated degradation. (A) Immunoblot of CFTR-3HA induced and chased as in Fig 3 with co-expressed Flag-Hsp70 or vector control, in the presence of siRNA against CHIP, HOP, or non-silencing (NS) siRNA. CHIP and HOP knockdown was verified by immunoblot and quantified. (B) Quantitations of CFTR bands B and C from (A) are shown relative to initial amounts, with CHIP knockdown, n = 3. Control data for CHIP knockdown from Fig 4C are shown here for comparison. (C) Quantitations of CFTR bands B and C from (A) are shown relative to initial amounts, with HOP knockdown, n = 3. Error bars show standard deviation from the mean, ** p<0.01, *** p<0.001.

We used siRNA to knock down either CHIP or HOP individually to 57% and 50% of non-silencing control, respectively, and performed induction-chase experiments. As reported above, in vector control conditions, CHIP knockdown increased band C, but interestingly depletion of HOP failed to alter CFTR band B and C levels (Fig 6A, 6B and 6C), suggesting that HOP plays a minimal role in degradation of CFTR. When Hsp70 was overexpressed, knockdown of CHIP was able to completely restore band C levels to those in vector controls (Fig 6A and 6B), whereas HOP knockdown had no effect (Fig 6A and 6C). This indicated that the lysosomal degradation of CFTR induced by Hsp70 was through CHIP, and that Hsp70 complexes with HOP and Hsp90 appear not to be involved. This role of Hsp70 is distinct from that regulated by DJA2.

### Inhibition of Hsp70 slows degradation of mature CFTR

**Fig 7:**
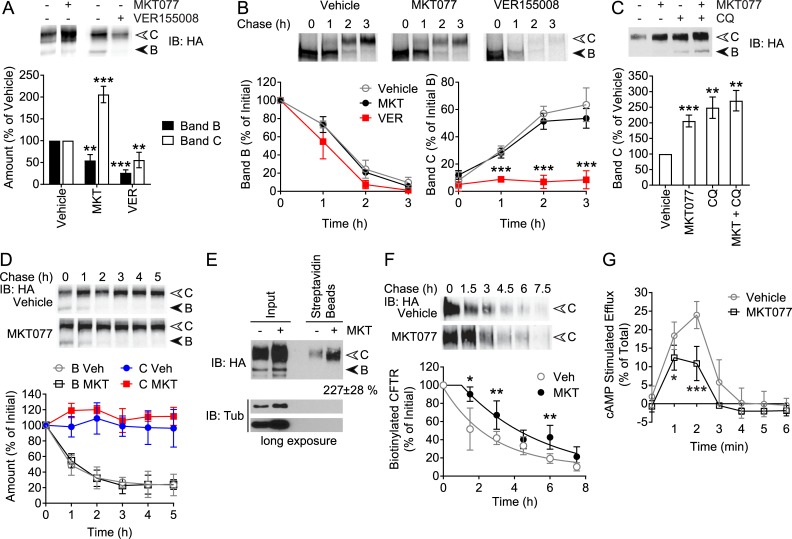
Hsp70 inhibitor MKT077 slows degradation of mature CFTR. (A) Immunoblot (IB) of CFTR-3HA stably expressed in HeLa cells treated with 10 μM MKT077, 50 μM VER155008 or vehicle control for 24 h. Quantitations of CFTR bands B and C are shown, relative to amounts of each in vehicle treated samples, n = 5. (B) Pulse-chase autoradiograph of CFTR as in Fig 1B, in the above cells, chased in the presence of 10 μM MKT077, 50 μM VER155008 or vehicle control. Quantitations of CFTR bands B and C are shown relative to initial amounts of band B, n = 3. (C) Immunoblot of cells treated as in (A) except with 10 μM MKT077, 100 μM CQ or both, or vehicle control. Quantitation of CFTR band C is shown relative to amounts in vehicle treated samples, n = 8 for MKT077, n = 4 for CQ with and without MKT077. (D) Immunoblot of CFTR-3HA in the above cells chased for 5 h in the presence of CHX and 10 μM MKT077 or vehicle control. Quantitations of CFTR bands B and C are shown relative to initial amounts of each, n = 5. (E) Cells were treated as in (A) with 10 μM MKT077 or vehicle control, cell surface biotinylated and pulled down with streptavidin beads. CFTR-3HA and tubulin control were detected by immunoblot in input lysate and biotinylated samples. CFTR band C in biotinylated samples was quantified relative to amounts in vehicle controls, n = 4. (F) The above cells were cell surface biotinylated without any treatment, then chased for 7.5 h in the presence of 10 μM MKT077 or vehicle control. Biotinylated CFTR-3HA was pulled down and detected by immunoblot as in (E). Quantitation of biotinylated CFTR band C is shown relative to the initial amount, with data fit to delayed one phase decay curves, n = 4. (G) Iodide efflux time courses of CFTR in the above cells treated with 10 μM MKT077 or vehicle control for 24 h. Cell surface CFTR was stimulated with cAMP cocktail and iodide efflux from cells was measured, relative to the total iodide released by detergent, n = 6. Error bars show standard deviation from the mean, * p<0.05, ** p<0.01, *** p<0.001.

The suppression of CFTR by Hsp70 suggested that it could be relieved by small molecule inhibitors of the chaperone. Among the established inhibitors, VER155008 is a direct competitor of ATP in the binding pocket of Hsp70 [36, 37] whereas MKT077 is an allosteric inhibitor with high affinity for the ADP-bound state of Hsp70 [41, 42]. We first evaluated the effect of these two inhibitors on CFTR expression levels under steady state conditions. Remarkably, MKT077 significantly increased band C levels almost two-fold over vehicle control, while somewhat decreasing band B levels (Fig 7A). In contrast, treatment with VER155008 led to a significant decrease in band C levels to around 40% of control, with band B levels decreased even more (Fig 7A). The kinetics of CFTR trafficking were next analyzed by pulse-chase experiments. VER155008 treatment almost completely abolished band C maturation with a slight reduction in band B levels, while MKT077 had no effect when compared to controls (Fig 7B). The data with the strong inhibitor VER155008 are consistent with the steady-state levels observed in Fig 7A, and indicate that Hsc70/Hsp70 is indeed required for efficient biosynthetic CFTR folding. In contrast, inhibition of Hsc70/Hsp70 by MKT077 may primarily affect mature CFTR, to prevent its turnover. This positive effect of MKT077 is consistent with the control of mature CFTR degradation by Hsp70 alone. MKT077 did not affect the interaction of CHIP with Hsp70 in immunoprecipitations (S4 Fig), and had no impact on cell viability under the conditions used (S5 Fig). The drug did not cause an HSR, and while a mild UPR was observed (S2A and S2B Fig), it did not change CFTR maturation kinetics (Fig 7B).

To establish that MKT077 affects mature CFTR, it was combined with CQ treatment. Additive effects of these inhibitors are expected when they act on independent mechanisms, but not if they block the same lysosomal degradation pathway. MKT077 and CQ increased band C levels by 2.2-fold and 2.5-fold, respectively, but the combination was not significantly more effective at 2.8-fold (Fig 7C). This result is consistent with MKT077 blocking lysosomal degradation of mature CFTR. The stability of total mature CFTR was addressed by CHX chase in the presence or absence of MKT077. Similar to induction-chase experiments, the levels of band C reflect the combined impact of anterograde trafficking and turnover of the mature protein. In vehicle controls, band C levels were fairly stable over a 5 h chase period (Fig 7D). Treatment with MKT077 led to a small increase in band C levels across all chase time points (Fig 7D), however this small effect may not account for the substantial increase observed at steady-state.

Band C comprises CFTR in all post-Golgi compartments. To quantify the PM population we used cell surface biotinylation. Cells treated with MKT077 or vehicle control were labelled with membrane-impermeable biotin, and CFTR pulled down on streptavidin beads. MKT077 increased cell surface CFTR by 2.27-fold (Fig 7E), in agreement with effects on total mature CFTR (Fig 7A). The biotinylation was specific for cell surface protein as shown by the lack of band B or tubulin in the pulldowns (Fig 7E). The turnover kinetics of PM CFTR were assessed by biotinylating the cell surface in the absence of MKT077, then adding drug and monitoring biotinylated CFTR over time. In vehicle controls, a significant amount of labelled WT CFTR is degraded during the 7.5 h chase period (Fig 7F). Treatment with MKT077 delayed the loss of biotinylated CFTR, with effects most clearly observed at the 1.5 h and 3 h time points (Fig 7F). The MKT077 data could only be fitted to a one-phase decay model by including a delay of around 1 h, whereas control data showed no delay. The half-life increased from 1.9 h for the control, to 3.2 h with MKT077. These results suggest that MKT077 blocks the ability of Hsp70 to target cell surface CFTR for degradation.

We next measured CFTR channel function in the presence or absence of MKT077 using iodide efflux assays. In vehicle control cells, CFTR displayed a maximal cAMP stimulated efflux response equivalent to 25% of the total iodide present in the cells, while treatment with MKT077 led to a reduction in channel function (Fig 7G). Although MKT077 clearly increases CFTR at the PM (Fig 7A and 7E), the decreased channel function suggests an accumulated pool of inactive CFTR at the cell surface.

**Fig 8:**
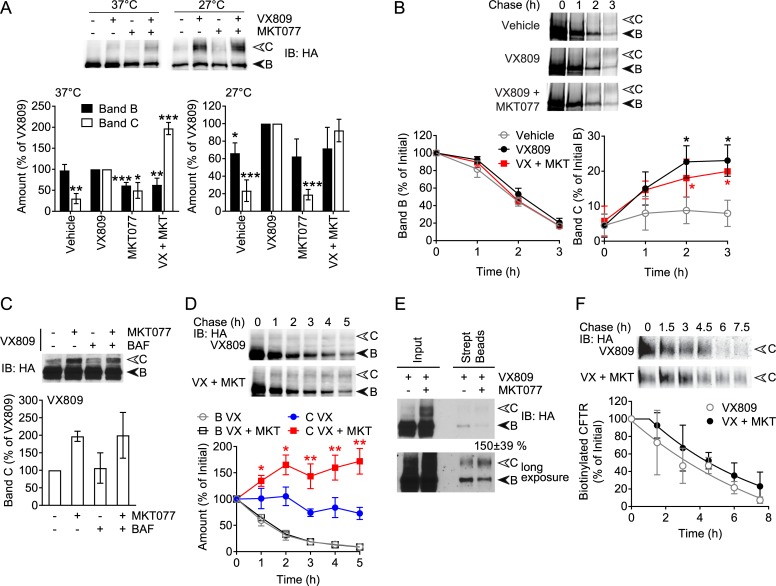
Combination of MKT077 with VX809 rescues ΔF508-CFTR expression. (A) Immunoblot (IB) of ΔF508-CFTR-3HA stably expressed in HeLa cells grown at either 37°C or the permissive temperature of 27°C and treated with 3 μM VX809, 10 μM MKT077 or both, or vehicle control for 24 h. Quantitations of CFTR bands B and C are shown relative to amounts of each in VX809 treated samples, n = 4 for MKT077, n = 9 for VX809 with and without MKT077. (B) Pulse-chase autoradiograph of ΔF508-CFTR in the above cells, chased in the presence of 3 μM VX809 with or without 10 μM MKT077, or vehicle control. Quantitations of CFTR bands B and C are shown relative to the initial amount of band B, n = 3. (C) The above cells were treated with 3 μM VX809, and either 10 μM MKT077, 100 nM Bafilomycin A1 (BAF) or both, or vehicle control, for 24 h. Quantitation of CFTR band C is shown relative to amounts in VX809 treated samples, n = 9 for MKT077, n = 4 for BAF with or without MKT077. (D) Immunoblot of ΔF508-CFTR in the above cells treated with 3 μM VX809 for 24 h and chased for 5 h in the presence of CHX and 3 μM VX809 and either 10 μM MKT077 or vehicle control. Quantitations of CFTR bands B and C are shown relative to initial amounts of each, n = 4. (E) The above cells were treated with 3 μM VX809 and either 10 μM MKT077 or vehicle control for 24 h, cell surface biotinylated and pulled down with streptavidin beads. CFTR-3HA and tubulin control were detected by immunoblot in input lysate and biotinylated samples. CFTR band C in biotinylated samples was quantified relative to amounts in vehicle controls, n = 3. (F) The above cells were grown at 27°C in the presence of 3 μM VX809 for 24 h, then cell surface biotinylated and chased at 37°C for 7.5 h in the presence of 3 μM VX809 and either 10 μM MKT077 or vehicle control. Biotinylated ΔF508-CFTR-3HA was pulled down and detected by immunoblot as in (E). Quantitation of biotinylated ΔF508-CFTR band C relative to the initial amount is shown, with data fit to delayed one phase decay curves, n = 4. Error bars show standard deviation from the mean, * p<0.05, ** p<0.01, *** p<0.001.

The increase in mature CFTR levels with MKT077 raises the possibility that it could be used to rescue the ΔF508 disease mutant. However, since the chaperone requirements for WT and ΔF508-CFTR are proposed to differ [16, 57], the effects on the WT channel could not be automatically presumed for the mutant. Correction of ΔF508-CFTR misfolding can be achieved either through growth at permissive temperatures of 27°C [58], or by treatment with pharmacological correctors such as VX809 [49]. We first compared the effect of MKT077 and VX809 treatment on HeLa cells stably expressing ΔF508-CFTR at 37°C. Treatment with VX809 rescued band C to approximately 35% of total, and this amount was set at 100% for comparison purposes (Fig 8A). As previously established, band C in untreated controls was around 7% of total, or 20% relative to the VX809 condition. MKT077 alone caused a small increase in band C to ~ 60% that with VX809 (Fig 8A). Interestingly, the combination of VX809 and MKT077 led to a synergistic increase in band C levels, to two-fold above that with VX809 alone (Fig 8A). We repeated the experiment under the permissive temperature, where similar to previous data, band C levels were approximately 20% of the total. Treatment with VX809 led to a large increase in band C levels and this was again set to 100%. Under these conditions, MKT077 alone or in combination with VX809 did not further increase the band C levels (Fig 8A).

To determine if the increase in band C levels upon combined treatment of VX809 and MKT077 is due to increased biosynthetic folding and trafficking, pulse-chase assays were performed. In untreated controls, barely any increase in band C was observed, and band B levels decreased rapidly, as expected. Treatment with VX809 promoted significant maturation of ΔF508-CFTR up to 25% of the initially radiolabeled material, whereas the combination of VX809 and MKT077 did not further increase CFTR maturation (Fig 8B). These results suggest that Hsp70 inhibition by MKT077 stabilizes rescued ΔF508-CFTR at a stage after initial biosynthesis. As a further test of this idea, MKT077 treatment was compared with inhibition of lysosomal degradation by the proton pump blocker Bafilomycin A1. Opposite to WT CFTR, the lysosome inhibitor had no effect on ΔF508-CFTR band C levels in the presence of VX809, and it did not affect the increase in band C caused by MKT077 (Fig 8C). These results suggest that only a minor fraction of VX809-stablilized mature ΔF508-CFTR was degraded in lysosomes, and likely would not account for the additional rescue caused by MKT077.

To assess the stability of rescued ΔF508-CFTR, it was expressed in the presence of VX809 at 37°C for 24 h, and chased with CHX in the presence of VX809 with or without MKT077. In controls with VX809 alone, band B levels were reduced rapidly and band C levels declined more slowly to around 75% of the starting material (Fig 8D). Notably, addition of MKT077 caused a steady increase in band C levels by more than 1.6-fold (Fig 8D). This increase must be due to increased maturation, but at slower time scales than in the pulse-chase (Fig 8B).

Next, the cell surface population of ΔF508-CFTR was examined. Treatment with MKT077 and VX809 resulted in a 1.5-fold increase in cell surface biotinylated band C, compared to VX809 alone (Fig 8E). Although the negative control tubulin was not detected, a small amount of band B was identified by biotin labelling (Fig 8E) which was most likely due to unconventional secretion from ER directly to PM bypassing the Golgi [59]. In support of this, a small amount of WT CFTR band B was also observed upon CQ treatment (Fig 7C). Unconventional secretion, however, could not explain the increase in Golgi-processed band C caused by MKT077 (Fig 8A, 8D and 8E). To measure turnover kinetics, ΔF508-CFTR was expressed with VX809 at the permissive temperature of 27°C in order to accumulate consistent amounts at the cell surface. Exposed CFTR was then biotin labeled and chased in the presence of VX809 with and without MKT077 at 37°C. MKT077 treatment modestly delayed the turnover of biotinylated ΔF508-CFTR compared to controls with only VX809 (Fig 8F). Fitting to a one-phase model showed a delay of around 1 h with MKT077, but the half-life was approximately the same as the control. Taken together, the results suggest that MKT077 inhibition of Hsp70 allows a population of ΔF508-CFTR to mature more slowly but more efficiently than the majority during biosynthesis (Fig 8B and 8D), eventually reaching the cell surface (Fig 8E), where its turnover may also be delayed (Fig 8F).

**Fig 9:**
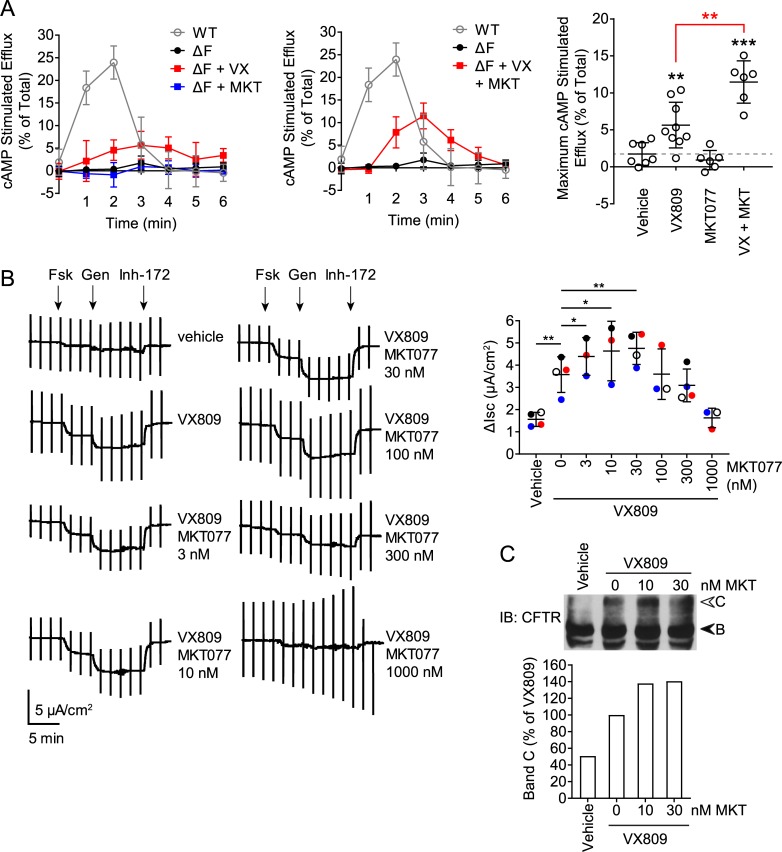
Combination of MKT077 with VX809 rescues ΔF508-CFTR function. (A) Iodide efflux time courses of ΔF508-CFTR stably expressed in HeLa cells treated with 3 μM VX809, 10 μM MKT077 or both or vehicle control for 24 h, as in Fig 7G,. Profiles of WT CFTR from Fig 7G are shown for comparison. Right, quantitation of the maximal cAMP stimulated efflux response of ΔF508-CFTR is shown, n = 9 for VX809, n = 6 for MKT077 with or without VX809. (B) Left, Ussing Chamber traces of CFBE41o^-^ cells stably expressing ΔF508-CFTR treated with 3 μM VX809 and increasing concentrations of MKT077, or vehicle. VX809 and MKT077 were present throughout the entire recording to ensure constant exposure of ΔF508-CFTR to the compounds. CFTR-associated currents were stimulated with forskolin (Fsk) and genistein (Gen) and inhibited with CFTR inhibitor-172 (Inh-172). Right, quantitation of the CFTR-associated short circuit current (ΔI_SC_) in the indicated conditions, relative to that with VX809 treatment, n = 4 with paired t-tests. (C) Cells as in (B) were grown on inserts and treated with 3 μM VX809 and increasing concentrations of MKT077, or vehicle. ΔF508-CFTR was detected by immunoblot (IB) and band C quantified relative to amounts in VX809 treated samples, n = 2. Error bars show standard deviation from the mean, * p<0.05, ** p<0.01, *** p<0.001.

We next addressed the effect of MKT077 on ΔF508-CFTR function. Using iodide efflux assays, we assessed the function of ΔF508-CFTR treated with VX809, MKT077, or a combination thereof for 24 h. For comparison, the data for WT CFTR (Fig 7G) are shown here again. Vehicle treated cells displayed minimal profiles of iodide efflux activity, and MKT077 alone had no effect (Fig 9A). Cells treated with VX809 displayed a moderate increase in efflux, that was further enhanced about two-fold when MKT077 was added in combination with VX809 (Fig 9A). Thus, unlike the WT channel, the increase in rescued ΔF508-CFTR with MKT077 results in greater channel function at the cell surface.

The experiments above were in model HeLa and HEK293 cells, and we wanted to examine MKT077 effects in an environment closer to that in patients. We therefore performed Ussing Chamber assays on CFBE41o^-^ bronchial epithelial cells stably expressing ΔF508-CFTR. The cells were cultured and polarized on inserts followed by treatment with VX809 and increasing concentrations of MKT077 for 24 h. VX809 and MKT077 were present in the bath solution during measurement to ensure that cell surface ΔF508-CFTR was exposed to a constant amount of drug. CFTR-associated currents were stimulated with forskolin and genistein, followed by inhibition with CFTR specific inhibitor 172 (Inh-172) [60–62]. VX809 treated samples displayed a larger stimulation of short circuit current (ΔI_SC_) of 3.6 μA/cm^2^ compared to vehicle treated samples at 1.6 μA/cm^2^ (Fig 9B). Titration of MKT077 from 3 nM to 1000 nM displayed a response curve with concentrations up to 30 nM displaying progressive increases in current up to a 35% above that with VX809 alone (Fig 9B), with higher concentrations displaying decreases in current. When the amounts of ΔF508-CFTR in the monolayer cells were examined, a consistent increase of about 40% in band C was observed with MKT077 compared to VX809 alone (Fig 9C). Thus, the positive effects of Hsp70 inhibition by MKT077 on rescued ΔF508-CFTR are observed in a bronchial epithelial cell line.

## Discussion

Taken together, our results outline how the Hsp70 chaperone system is balanced between the folding and degradation of immature and mature CFTR. The functional amounts of CFTR depend on the expression levels of specific chaperone components (S6 Fig). In agreement with previous reports [25], DJA1 primarily supports the folding of immature CFTR (Fig 2). In contrast, DJA2 has a unique role in inducing the degradation of immature CFTR, through J domain stimulation of Hsc70/Hsp70, that promotes chaperone-CHIP complexes with CFTR (Figs 2, 3 and 4). Although Hsc70/Hsp70 acts both in CFTR folding and degradation, its net effect is to suppress maturation (Fig 1), and promote lysosomal degradation of mature CFTR through CHIP (Figs 5 and 6). Allosteric inhibition of Hsc70/Hsp70 by MKT077 enhances the population of mature CFTR and ΔF508-CFTR, by slowing turnover and allowing delayed maturation, respectively (Figs 7 and 8). For ΔF508-CFTR, pharmacologic targeting of Hsc70/Hsp70 results in a partial rescue of its channel function (Fig 9). The positive effects of MKT077 are consistent with it acting on the net degradative function Hsc70/Hsp70, comparable to the experiments manipulating expression (Figs 1 and 5), while complete inhibition with the ATP competitor VER155008 primarily blocks folding (Fig 7).

Our studies suggest that efficient rescue of ΔF508-CFTR can best be achieved by strategies targeting different mechanisms. The multiple barriers impeding ΔF508-CFTR include trafficking, function and biological stability. Correctors like VX809 (lumacaftor) target the trafficking defect of ΔF508-CFTR, while potentiators like VX770 (ivacaftor) increase channel function by acting on the gating mechanism of CFTR mutants. Current clinical treatment uses a combination of these two compounds [51–53]. Our studies demonstrate that the stability barrier of rescued ΔF508-CFTR can be circumvented by a new combination of mechanisms. While VX809 corrects the trafficking defect of ΔF508-CFTR, MKT077 allows the further accumulation of the rescued protein. MKT077 appears to increase the stability of ΔF508-CFTR against ERAD, relieving the suppression of maturation by Hsc70/Hsp70 over longer time scales. WT CFTR escapes ERAD more efficiently, and MKT077 increases its stability at the cell surface. In prinicple, MKT077 can affect Hsc70/Hsp70-mediated degradation of CFTR both during and after maturation. This raises the possibility that MKT077 treatment may also be effective against other misfolding CF mutations such as R560T, A561E, R1066C, N1303K (http://www.cftr2.org/) [52].

The degradation role of Hsp70 has important implications for the understanding of CF. The knockdown and VER155008 data suggest that only a fraction of endogenous Hsc70 and Hsp70 is necessary for CFTR folding and trafficking, supported by endogenous levels of DJA1 and DJA2. Because Hsc70 and Hsp70 are highly abundant in cells [63, 64], their levels are unlikely to be limiting for the folding of ΔF508-CFTR. Instead, the excess endogenous Hsc70/Hsp70 appears to naturally restrict the population of mature CFTR by favouring lysosomal degradation. Moreover, excess Hsc70/Hsp70 promotes ERAD when stimulated by DJA2 and the ER-associated co-chaperones CSPα and DNAJB12 [25, 27, 28]. Unlike these membrane-associated DNAJs, DJA2 is only loosely attached to membranes by prenylation, which is not essential for some of its folding and ERAD functions [30]. DJA2 may thus interact with parts of CFTR that are farther from the membrane.

Hsc70/Hsp70 is a promising drug target for cancer therapies, and there has been recent progress in developing novel small molecules that modulate different aspects of its mechanism. The use of such an agent at low doses to achieve partial inhibition as evidenced by our studies with MKT077 could be a promising therapeutic approach to preserve ΔF508-CFTR at the PM in CF patients. An interesting question is how VER155008 and MKT077 have opposing effects on CFTR. As an ATP competitor, VER155008 provides a strict block of the Hsc70 ATPase and thus appears to act only as an inhibitor, whereas the allosteric inhibition of MKT077 modulates the ATPase cycle. In this way, MKT077 may preferentially affect Hsc70 function with CHIP before the pro-folding activities of the chaperone. CHIP activity depends on the ATPase cycling rate of Hsc70 [18], therefore specific binding of MKT077 to the ADP state [42] could modulate this rate to impair CHIP. Although the Hsc70 interface with CHIP is also allosterically regulated [19, 65, 66], MKT077 did not affect CHIP binding, suggesting that the drug does not target the interface. In CFBE41o^-^ cells, MKT077 rescue of band C is observed at lower concentrations than in HeLa cells, possibly because Hsc70/Hsp70 levels are also lower in the bronchial epithelial cells. The properties of MKT077 derivatives that are optimal for cancer therapies [43] may not be the same as those required for CF treatment. Finally, it is uncertain whether specific pharmacologic targeting of the Hsc70-CHIP interaction is possible due to the charged nature of the interaction [65, 66].

DJA1 and DJA2 are highly homologous (69% similarity) but there is growing evidence they have distinct biological functions. The co-chaperones differ in certain biochemical properties, which underlies their different abilities to support Hsc70-mediated folding of various proteins [30, 67, 68]. We demonstrated that DJA1 but not DJA2 was required for maturation of the hERG channel [29]. DJA1 is also specifically required for Activation-Induced Deaminase [69], and for proper regulation of the Androgen Receptor [70]. Conversely, we found that DJA2 but not DJA1 is necessary for luciferase refolding [30]. DJA2 is also known to be important for signaling through certain trimeric G proteins [71]. With CFTR, DJA1 and DJA2 have different roles at the ER and cell surface. DJA1 promotes CHIP-mediated ubiquitination [24], but its net effect favours biosynthetic folding, while DJA2 promotes ERAD. At the PM, these roles are reversed, with DJA1 promoting internalization and lysosomal degradation of misfolded CFTR while DJA2 assists its refolding by Hsc70 [31, 34]. It is possible that different conformational states of partially folded CFTR are recognized by the DNAJs at the ER and PM, leading to distinct folding or degradation regimes. Also, like the DNAJs, MKT077 modulates the ATPase cycle of Hsc70/Hsp70 to shift the net balance of folding and degradation.

## Materials and methods

### Cell Lines and Plasmids

HeLa cells stably expressing wild-type (WT) CFTR-3HA or ΔF508-CFTR-3HA were provided by Dr. G.L. Lukacs [31]. ΔF508-CFBE41o^-^ were as published [62]. HEK293 and HEK293-Tet-On cells were purchased from ATCC (CRL-1573) and Clontech (631183), respectively.

The following constructs were inserted into pcDNA3.1 vector backbone: Flag-Hsp70, myc-DNAJA1, myc-DNAJA2, and DJA2ΔJ-myc [30]. For HEK293-Tet-On experiments CFTR-3HA was inserted into the pTRE-Tight vector. Plasmid pcDNA3.1-CFTR-3HA was provided by Dr. D. Y. Thomas and pEGFP-C1-HA-RhoB was provided by Dr. D. Pérez-Sala.

The following siRNA constructs were purchased from Dharmacon: Non-silencing siRNA (D-001810); ON-TARGETplus SMARTpool *HSPA1A* (L-005168), *HSPA1B* (L-003501), *HSPA8* (L-017609), *DNAJA1* (L-019617), *DNAJA2* (L-012104), HOP/*STIP1* (L-019802), CHIP/*STUB1* (L-007201).

### Reagents and Antibodies

Anti-Hsp70/Hsc70 (SMC-104), anti-Hsp90 total, 4F3.E8 (SMC-149) and anti-DJB1, 3B9.E6 (SMC-145D) were purchased from StressMarq. Anti-CHIP (C9243), anti-Flag M2 (F1804) were from Sigma-Aldrich. Anti-DJA1, KA2A5.6 (sc-59554), anti-myc (SC-40) and anti-RhoB (SC-180) were from Santa Cruz Biotechnology. Anti-HA.11 clone 16B12 (901501) was from BioLegend. CFTR antibodies M3A7 (MAB3480) and L12B4 (MAB3484) were from EMD Millipore. Anti-HOP (DS14F5) was from Enzo Lifesciences. Anti-BiP, clone 40/BiP (610979) was from BD Biosciences, and anti-CHOP, 9C8 (MA1-250) was from ThermoFisher Scientific. Anti-DJA2 antibodies were raised in rabbits as previously described [67].

All chemicals were purchased from Sigma-Aldrich unless otherwise specified. Compounds MKT077 (4621) and VER155008 (3803) were purchased from Tocris Bioscience. VX809 (S1565) was purchased from Selleckchem. Apoptozole (17675) was purchased from Cayman Chemicals. Bafilomycin A1 (10–2060) was purchased from Focus Biomolecules. FLAG peptide (DK-8) (LT12022) was purchased from LifeTein.

### Cell culture and transfection

HeLa and HEK293 cells were cultured in DMEM, high glucose and glutamine (Gibco-Invitrogen), supplemented with 10% fetal bovine serum, 1 mM sodium pyruvate, 100 units/ml Penicillin and 100 μg/ml Streptomycin. The cells were cultured in 5% CO_2_ and unless otherwise stated, at 37°C.

For siRNA transfection, HeLa or HEK293 cells were seeded at 2.5 × 10^6^ and 5 × 10^6^ per 100 mm dish, respectively. The following day, cells were transfected with 100 nM siRNA and 5 μL DharmaFECT1 Transfection Reagent (ThermoFisher Scientific, Cat. T-2001-03) per 100 mm dish according to manufacturer protocol in DMEM supplemented with FBS. For plasmid transfection, HEK cells were transfected with 24 μg total DNA of pcDNA3.1 WT CFTR-3HA and Hsp70 or co-chaperone (DJA1, DJA2, DJA2ΔJ) in a 2:1 ratio, and 24 μL of Lipofectamine 2000 transfection reagent (ThermoFisher Scientific, Cat. 11668019) per 100 mm dish according to the manufacturer’s protocol in DMEM supplemented with FBS. Cells were maintained in the 100 mm dishes, or trypsinized 6 hours after siRNA or plasmid transfection and distributed into 5 x 60 mm dishes. Cells were grown for 48 hours prior to experiments.

For qPCR analysis, HEK293 cells were seeded at 1 × 10^6^ per well into a 6-well plate. The following day, the cells were transfected with 4 μg total DNA of pcDNA3.1 WT CFTR-3HA and co-chaperone or empty-vector in a 2:1 ratio and with 4 μL of Lipofectamine 2000 transfection reagent. The cells were grown for 48 hours, and total RNA was isolated using Trizol Reagent (ThermoScientific, Cat. 15596026). 1 μg total RNA was reverse-transcribed using the QuantiTect Reverse Transcription Kit (Qiagen, Cat. 205311), then the cDNA was cycled using the QuantiFast SYBR Green PCR Kit (Qiagen, Cat. 204054) in the MX3005P QPCR system (Agilent). The primers used for the qPCR were as follows: CFTR, 5’-GGAAAGAGAATGGGATAGAGAGCTGGC-3’ and 5’-CCGGGTCATAGGAAGCTATGATTCTTCCCAG-3’; beta actin, 5’-’CCTGGCACCCAGCACAATAAG-3’ and 5’-AAGTCATAGTCCGCCTAGAAGC-3’.

For viability measurements, HeLa cells stably expressing CFTR or ΔF508-CFTR were treated with vehicle, or 10 μM MKT077, or or 1 μM staurosporine for 24 hours in 24-well plates. Cell viability was assessed with AlamarBlue Cell Viability Reagent (Thermofisher Scientific, Cat. DAL1025) according to the manufacturer’s instructions. Briefly, cells were incubated with 500 μl Alamar Blue reagent diluted 1/10 in complete medium, for 2 hours at 37°C in 5% CO_2_ and then fluorescence was measured (excitation λ 560 nm, emission λ 590 nm) in a SynergyMX plate reader, and normalized to protein amounts measured by the BCA Protein Assay Kit (ThermoFisher Scientific, Cat. 23225).

### Cell lysates

Cells were lysed in RIPA buffer containing 150 mM NaCl, 20 mM Tris-Cl, 1% TX-100, 0.1% SDS, 0.5% sodium deoxycholate and Complete EDTA-free protease inhibitor (Sigma Aldrich, Cat. 11873580001). Soluble material was separated by centrifugation at 20,000 x g for 5 min at 4°C. CFTR and chaperones/co-chaperones were separated on 6% and 10% SDS-PAGE, respectively. Protein concentrations were normalized for immunoblots after estimation using the Pierce BCA Protein Assay kit. Immunoblots were detected using chemiluminescence and a FluorChem HD2 digital camera (AlphaInnotech) in its linear detection range, and densitometry was quantified using ImageJ 1.46r software.

### Metabolic pulse-chase

Metabolic labelling assays were performed as previously described [5] with the following modifications. Cells were serum starved in DMEM without methionine and cysteine (ThermoFisher Scientific, Cat. 21013024) for 45 min, and pulse-labelled in 100 μCi/mL EasyTag™ Express Protein Labeling Mix, [35S] (Perkin Elmer, Cat. NEG772) for 15 min (WT CFTR) or 1 hour (ΔF508-CFTR). Cells were rinsed with cold PBS containing 1mM MgCl_2_ and 0.1mM CaCl_2_ and chased in DMEM supplemented with 10% FBS, 2 mM glutamine, 2 mM methionine and 2 mM cysteine. Cells were harvested at 0, 1, 2, or 3 h in 500 μL RIPA buffer. Soluble material was separated by centrifugation at 20,000 x g for 5 min at 4°C. Protein concentrations were normalized using the BCA Protein Assay Kit, and pulse-labelled CFTR was isolated by immunoprecipitation with a mixture of CFTR monoclonal antibodies (Millipore, MAB3480 (1.5 μg) and MAB3484 (1.5 μg) per sample) and 50 μL protein G agarose beads (Millipore, Cat: 16–266) for 2.5 hours at 4°C. Beads were washed 3 times in RIPA buffer and proteins were eluted in Laemmli loading buffer. CFTR bands were separated by 6% SDS-PAGE and radioactive bands were detected using a Typhoon scanner (GE Healthcare), then quantified using ImageJ 1.46r software.

### Induction and cycloheximide chase

HEK293-Tet-On cells were grown in 100 mm poly-lysine coated dishes and transfected with plasmid as above. For experiments requiring siRNA transfection it was carried out prior to DNA transfection as above, with the exception that cells were distributed into 4 wells of a 6-well plate after siRNA transfection. The following day cells were transfected with pTRE-WT-CFTR and indicated chaperone in a 2:1 ratio as above. One day after plasmid transfection, CFTR expression was induced by 1 μg/mL doxycycline for 6 h. Cells were chased in 50 μg/mL cycloheximide (CHX) in the presence of 0.1% DMSO vehicle control, 20 μM MG132 or 200 μM chloroquine (CQ), and analyzed by immunoblot.

HeLa cells stably expressing WT CFTR or ΔF508-CFTR were seeded in 6-well plates at 2 x 10^5^ cells per well, and grown for 24 h in the presence of vehicle or 3 μM VX809, respectively. Cells were chased in 50 μg/mL CHX in the presence of vehicle or 10 μM MKT077, and analyzed by immunoblot.

### Cell surface biotinylation

HeLa cells stably expressing WT CFTR or ΔF508-CFTR were seeded at 5 x 10^5^ cells per 60 mm dish. For steady-state experiments, cells expressing ΔF508-CFTR were treated with 3 μM VX809 and vehicle or 10 μM MKT077, and cells expressing CFTR were treated with vehicle or 10 μM MKT077, at 37°C for 24 h. Cell surface CFTR was labelled with 1 mg/mL EZ-Link Sulfo-N-hydroxysulfosuccinimide-SS-Biotin (ThermoFisher Scientific, Cat. 21331) on ice for 30 minutes, then excess biotin was quenched with 1% BSA. Cells were lysed in modified RIPA buffer and protein concentrations were normalized as above. Biotinylated proteins were pulled down with streptavidin-agarose beads (ThermoFisher Scientific, Cat. 20347) for 1 h at 4°C, eluted with Laemmli loading buffer, and CFTR was detected by immunoblot. For chase experiments, cells expressing ΔF508-CFTR were treated with 3 μM VX809 and grown at 27°C for 24 h; cells expressing CFTR were grown at 37°C for 24 h. Cell surface CFTR was biotinylated and quenched as above, and samples chased at 37°C over a period of 7.5 h in the presence of vehicle or 10 μM MKT077. Data were fit to a delayed one-phase decay model using GraphPad Prism 7.03.

### Immunoprecipitation

HEK-Tet-On cells in 100 mm dishes were transfected with pTRE-WT-CFTR and vector, or Flag-Hsp70 without and with DJA2 as above. CFTR was induced for 6 h in the presence of 20 μM MG132. Cells were lysed in PBS containing 1% TX-100, and protein concentrations were normalized using the BCA protein assay. Hsp70 was then immunoprecipitated with anti-Flag M2 magnetic beads (Sigma, Cat. M8823) for 2 hrs at 4°C. Beads were washed 3 times with PBS containing 0.1% TX-100, the proteins were eluted with 1 mg/mL FLAG peptide (DK-8), and then analyzed by immunoblot.

### Iodide efflux

Experiments were performed as previously described [7]. Briefly, HeLa cells stably expressing WT CFTR or ΔF508-CFTR were seeded in 6 well plates at 2 x 10^5^ cells per well. Cells were treated with 0.1% DMSO as vehicle control, 3 μM VX809, 10 μM MKT077 or both compounds for 24 h at 37°C. Cells were loaded with NaI in efflux buffer containing 3 mM KNO_3_, 2 mM Ca(NO_3_)_2_, 11 mM glucose and 20 mM HEPES pH 7.5, supplemented with 136 mM NaI, for 1 h. Excess iodide was removed with several washes of efflux buffer supplemented with 136 mM NaNO_3_ and no iodide. To stimulate cell surface CFTR channel activity, intracellular cAMP levels were raised by addition of 10 μM forskolin,1mM isobutyl methyl xanthine, and 100 μM 8-(4-Chlorophenylthio) adenosine 3′,5′-cyclic monophosphate sodium salt. Total cellular iodide content was measured after permeabilizing cell membranes in 1% TX-100. An iodide sensitive electrode was used to measure iodide flux.

### Ussing chamber measurements

ΔF508-CFBE41o^-^ (80,000 cells) were seeded onto fibronectin-coated Transwells 6.5mm inserts (Corning Incorporated, Life Science, New York) and the apical medium was removed 24 h later to establish an air-liquid interface. After three days, the basolateral medium was replaced by OptiMEM supplemented with 2% FBS and the next day to OptiMEM with no FBS. The monolayers were treated on both sides with OptiMEM with no FBS and compounds at the desired concentration for 24 h before being either sampled for immunoblots, or mounted in EasyMount chambers and voltage clamped using a VCCMC6 multichannel current-voltage clamp (Physiologic Instruments, San Diego, CA). The apical membrane conductance was functionally isolated by permeabilizing the basolateral membrane with 200 μg/ml nystatin and imposing an apical-to-basolateral Cl^-^ gradient. The basolateral bathing solution contained 1.2 mM NaCl, 115 mM Na-gluconate, 25 mM NaHCO_3_, 1.2 mM MgCl_2_, 4 mM CaCl_2_, 2.4 mM KH_2_PO_4_, 1.24 mM K_2_HPO_4_ and 10 mM glucose, pH 7.4. The CaCl_2_ concentration was increased to 4 mM to compensate for the chelation of calcium by gluconate. The apical bathing solution contained 115 mM NaCl, 25 mM NaHCO_3_, 1.2 mM MgCl_2_, 1.2 mM CaCl_2_, 2.4 mM KH_2_PO_4_, 1.24 mM K_2_HPO_4_ and 10 mannitol, pH 7.4. The apical solution contained mannitol instead of glucose to eliminate currents mediated by Na^+^-glucose co-transport. Successful permeabilization of the basolateral membrane was obvious from the reversal of I_SC_ under these conditions. Solutions were continuously gassed and stirred with 95% O_2_-5% CO_2_ and maintained at 37°C. Ag/AgCl reference electrodes were used to measure transepithelial voltage and pass current. Pulses (1 mV amplitude, 1 s duration) were delivered every 90 s to monitor resistance. The voltage clamps were connected to a PowerLab/8SP interface for data collection. CFTR was activated by adding 10 μM forskolin and 50 μM genistein to the apical bathing solution. CFTR associated current was inhibited by addition of 10 μM CFTR inhibitor-172.

## Supporting information

S1 Fig No effect of lysosome inhibitor on pulse-chase. CFTR-3HA was stably expressed in HeLa cells transfected with siRNA against Hsc70 and Hsp70 (si-Hsc/p70) or non-silencing (NS) siRNA. Pulse-chase autoradiograph of CFTR-3HA is shown, with quantitations of bands B and C relative to initial amounts of band B, n = 3. Error bars show standard deviation from the mean.
(PDF)

S2 Fig Analysis of stress responses. (A) Induction of chaperones. HeLa cells stably expressing CFTR-3HA were transfected with siRNA against Hsc70 and Hsp70 (si-Hsc/p70), or DJA1 (si-DJA1), or DJA2 (si-DJA2), or non-silencing (NS) siRNA, or 10 μM MKT077 or vehicle control for 24 h. The indicated chaperones were detected by immunoblot, and quantitations relative to non-silencing or vehicle controls are shown; si-Hsc/p70 n = 3; si-DJA1, si-DJA2 and MKT077, n = 4. (B) Induction of CHOP. Cells were transfected with siRNA as above, or treated with 0.1 μM thapsigargin (TG), or 10 μM MKT077, or vehicle control for 24 h. CHOP was detected by immunoblot, and quantitations relative to maximum induction by thapsigargin are shown; si-Hsc/p70, si-DJA1 and si-DJA2, n = 2; MKT077, n = 4. Error bars show standard deviation from the mean.
(PDF)

S3 Fig Measurement of mRNA. HEK293 cells were transfected with CFTR-3HA and myc-DJA1, myc-DJA2 or vector control. Total mRNA was extracted and reverse-transcribed, amounts of CFTR cDNA were determined by quantitative PCR normalized to actin cDNA. Quantitations relative to amounts in vector controls are shown, n = 5. Error bars show standard deviation from the mean, * p<0.05.
(PDF)

S4 Fig MKT077 does not affect Hsp70-CHIP interaction. HeLa cells stably expressing CFTR-3HA were transfected with Flag-Hsp70 or empty vector, and treated with MKT077 or vehicle control. Flag-Hsp70 was immunoprecipitated (IP) and bound CHIP detected by immunoblot (IB). Quantified CHIP was normalized to total expression and amount of Hsp70 in the IP, and shown as a percentage of the IP with Flag-Hsp70 and vehicle control.
(PDF)

S5 Fig Cell viability. HeLa cells stably expressing CFTR-3HA or ΔF508-CFTR-3HA were treated with 10 μM MKT077, or 1 μM staurosporine (STS), or vehicle control for 24 hours. Viability was determined by fluorescence of Alamar blue reagent, normalized to total protein amounts. Quantitations relative to vehicle controls are shown, n = 4. Error bars show standard deviation from the mean.
(PDF)

S6 Fig Model of CFTR degradation regulated by DNAJA2 and Hsp70. At the ER, DNAJA1 assists the folding of immature CFTR. Excess DNAJA2 and Hsp70 promote ERAD of CFTR through the E3 ligase CHIP. At the PM, Hsp70 promotes the degradation of mature CFTR in lysosomes, also through CHIP. Hsp70 inhibitor MKT077 increases mature CFTR by inhibiting its degradation. MKT077 also increases mature ΔF508-CFTR by allowing its slow maturation and accumulation.
(PDF)

## References

[1] Amaral MD. Processing of CFTR: traversing the cellular maze—how much CFTR needs to go through to avoid cystic fibrosis? Pediatr Pulmonol. 2005;39(6):479–91. 10.1002/ppul.20168 . 15765539

[2] Sheppard DN, Welsh MJ. Structure and function of the CFTR chloride channel. Physiol Rev. 1999;79(1 Suppl):S23–45. 10.1152/physrev.1999.79.1.S23 . 9922375

[3] Ward CL, Kopito RR. Intracellular turnover of cystic fibrosis transmembrane conductance regulator. Inefficient processing and rapid degradation of wild-type and mutant proteins. J Biol Chem. 1994;269(41):25710–8. . 7523390

[4] Riordan JR, Rommens JM, Kerem B, Alon N, Rozmahel R, Grzelczak Z, et al Identification of the cystic fibrosis gene: cloning and characterization of complementary DNA. Science. 1989;245(4922):1066–73. 10.1126/science.2475911 . 2475911

[5] Lukacs GL, Mohamed A, Kartner N, Chang XB, Riordan JR, Grinstein S. Conformational maturation of CFTR but not its mutant counterpart (delta F508) occurs in the endoplasmic reticulum and requires ATP. EMBO J. 1994;13(24):6076–86. 7529176 10.1002/j.1460-2075.1994.tb06954.x PMC395586

[6] Du K, Sharma M, Lukacs GL. The DeltaF508 cystic fibrosis mutation impairs domain-domain interactions and arrests post-translational folding of CFTR. Nat Struct Mol Biol. 2005;12(1):17–25. 10.1038/nsmb882 . 15619635

[7] Du K, Lukacs GL. Cooperative assembly and misfolding of CFTR domains in vivo. Mol Biol Cell. 2009;20(7):1903–15. 10.1091/mbc.E08-09-0950 19176754 PMC2663924

[8] Kim SJ, Skach WR. Mechanisms of CFTR Folding at the Endoplasmic Reticulum. Front Pharmacol. 2012;3:201 10.3389/fphar.2012.00201 23248597 PMC3521238

[9] Rabeh WM, Bossard F, Xu H, Okiyoneda T, Bagdany M, Mulvihill CM, et al Correction of both NBD1 energetics and domain interface is required to restore DeltaF508 CFTR folding and function. Cell. 2012;148(1–2):150–63. 10.1016/j.cell.2011.11.024 22265408 PMC3431169

[10] Loo MA, Jensen TJ, Cui L, Hou Y, Chang XB, Riordan JR. Perturbation of Hsp90 interaction with nascent CFTR prevents its maturation and accelerates its degradation by the proteasome. EMBO J. 1998;17(23):6879–87. 10.1093/emboj/17.23.6879 9843494 PMC1171036

[11] Wang X, Venable J, LaPointe P, Hutt DM, Koulov AV, Coppinger J, et al Hsp90 cochaperone Aha1 downregulation rescues misfolding of CFTR in cystic fibrosis. Cell. 2006;127(4):803–15. 10.1016/j.cell.2006.09.043 . 17110338

[12] Young JC. The role of the cytosolic HSP70 chaperone system in diseases caused by misfolding and aberrant trafficking of ion channels. Dis Model Mech. 2014;7(3):319–29. 10.1242/dmm.014001 24609033 PMC3944492

[13] Kampinga HH, Craig EA. The HSP70 chaperone machinery: J proteins as drivers of functional specificity. Nat Rev Mol Cell Biol. 2010;11(8):579–92. 10.1038/nrm2941 20651708 PMC3003299

[14] Hartl FU, Bracher A, Hayer-Hartl M. Molecular chaperones in protein folding and proteostasis. Nature. 2011;475(7356):324–32. 10.1038/nature10317 . 21776078

[15] Meacham GC, Lu Z, King S, Sorscher E, Tousson A, Cyr DM. The Hdj-2/Hsc70 chaperone pair facilitates early steps in CFTR biogenesis. EMBO J. 1999;18(6):1492–505. 10.1093/emboj/18.6.1492 10075921 PMC1171238

[16] Coppinger JA, Hutt DM, Razvi A, Koulov AV, Pankow S, Yates JR 3rd, et al A chaperone trap contributes to the onset of cystic fibrosis. PLoS One. 2012;7(5):e37682 10.1371/journal.pone.0037682 22701530 PMC3365120

[17] Meacham GC, Patterson C, Zhang W, Younger JM, Cyr DM. The Hsc70 co-chaperone CHIP targets immature CFTR for proteasomal degradation. Nat Cell Biol. 2001;3(1):100–5. 10.1038/35050509 . 11146634

[18] Matsumura Y, David LL, Skach WR. Role of Hsc70 binding cycle in CFTR folding and endoplasmic reticulum-associated degradation. Mol Biol Cell. 2011;22(16):2797–809. 10.1091/mbc.E11-02-0137 21697503 PMC3154877

[19] Matsumura Y, Sakai J, Skach WR. Endoplasmic reticulum protein quality control is determined by cooperative interactions between Hsp/c70 protein and the CHIP E3 ligase. J Biol Chem. 2013;288(43):31069–79. 10.1074/jbc.M113.479345 23990462 PMC3829420

[20] Younger JM, Chen L, Ren HY, Rosser MF, Turnbull EL, Fan CY, et al Sequential quality-control checkpoints triage misfolded cystic fibrosis transmembrane conductance regulator. Cell. 2006;126(3):571–82. 10.1016/j.cell.2006.06.041 . 16901789

[21] Morito D, Hirao K, Oda Y, Hosokawa N, Tokunaga F, Cyr DM, et al Gp78 cooperates with RMA1 in endoplasmic reticulum-associated degradation of CFTRDeltaF508. Mol Biol Cell. 2008;19(4):1328–36. 10.1091/mbc.E07-06-0601 18216283 PMC2291415

[22] Wang B, Heath-Engel H, Zhang D, Nguyen N, Thomas DY, Hanrahan JW, et al BAP31 interacts with Sec61 translocons and promotes retrotranslocation of CFTRDeltaF508 via the derlin-1 complex. Cell. 2008;133(6):1080–92. 10.1016/j.cell.2008.04.042 . 18555783

[23] Dalal S, Rosser MF, Cyr DM, Hanson PI. Distinct roles for the AAA ATPases NSF and p97 in the secretory pathway. Mol Biol Cell. 2004;15(2):637–48. 10.1091/mbc.E03-02-0097 14617820 PMC329284

[24] Younger JM, Ren HY, Chen L, Fan CY, Fields A, Patterson C, et al A foldable CFTR{Delta}F508 biogenic intermediate accumulates upon inhibition of the Hsc70-CHIP E3 ubiquitin ligase. J Cell Biol. 2004;167(6):1075–85. 10.1083/jcb.200410065 15611333 PMC2172621

[25] Grove DE, Fan CY, Ren HY, Cyr DM. The endoplasmic reticulum-associated Hsp40 DNAJB12 and Hsc70 cooperate to facilitate RMA1 E3-dependent degradation of nascent CFTRDeltaF508. Mol Biol Cell. 2011;22(3):301–14. 10.1091/mbc.E10-09-0760 21148293 PMC3031462

[26] Farinha CM, Nogueira P, Mendes F, Penque D, Amaral MD. The human DnaJ homologue (Hdj)-1/heat-shock protein (Hsp) 40 co-chaperone is required for the in vivo stabilization of the cystic fibrosis transmembrane conductance regulator by Hsp70. Biochem J. 2002;366(Pt 3):797–806. 10.1042/BJ20011717 12069690 PMC1222832

[27] Schmidt BZ, Watts RJ, Aridor M, Frizzell RA. Cysteine string protein promotes proteasomal degradation of the cystic fibrosis transmembrane conductance regulator (CFTR) by increasing its interaction with the C terminus of Hsp70-interacting protein and promoting CFTR ubiquitylation. J Biol Chem. 2009;284(7):4168–78. 10.1074/jbc.M806485200 19098309 PMC2640980

[28] Yamamoto YH, Kimura T, Momohara S, Takeuchi M, Tani T, Kimata Y, et al A novel ER J-protein DNAJB12 accelerates ER-associated degradation of membrane proteins including CFTR. Cell Struct Funct. 2010;35(2):107–16. Epub 2010/12/15. JST.JSTAGE/csf/10023 [pii]. . 21150129 10.1247/csf.10023

[29] Walker VE, Wong MJ, Atanasiu R, Hantouche C, Young JC, Shrier A. Hsp40 chaperones promote degradation of the HERG potassium channel. J Biol Chem. 2010;285(5):3319–29. 10.1074/jbc.M109.024000 19940115 PMC2823420

[30] Baaklini I, Wong MJ, Hantouche C, Patel Y, Shrier A, Young JC. The DNAJA2 substrate release mechanism is essential for chaperone-mediated folding. J Biol Chem. 2012;287(50):41939–54. 10.1074/jbc.M112.413278 23091061 PMC3516741

[31] Okiyoneda T, Barriere H, Bagdany M, Rabeh WM, Du K, Hohfeld J, et al Peripheral protein quality control removes unfolded CFTR from the plasma membrane. Science. 2010;329(5993):805–10. 10.1126/science.1191542 . 20595578 PMC5026491

[32] Scheufler C, Brinker A, Bourenkov G, Pegoraro S, Moroder L, Bartunik H, et al Structure of TPR domain-peptide complexes: critical elements in the assembly of the Hsp70-Hsp90 multichaperone machine. Cell. 2000;101(2):199–210. 10.1016/S0092-8674(00)80830-2 . 10786835

[33] Li J, Soroka J, Buchner J. The Hsp90 chaperone machinery: conformational dynamics and regulation by co-chaperones. Biochim Biophys Acta. 2012;1823(3):624–35. 10.1016/j.bbamcr.2011.09.003 . 21951723

[34] Bagdany M, Veit G, Fukuda R, Avramescu RG, Okiyoneda T, Baaklini I, et al Chaperones rescue the energetic landscape of mutant CFTR at single molecule and in cell. Nat Commun. 2017;8(1):398 10.1038/s41467-017-00444-4 28855508 PMC5577305

[35] Williams DR, Ko SK, Park S, Lee MR, Shin I. An apoptosis-inducing small molecule that binds to heat shock protein 70. Angew Chem Int Ed Engl. 2008;47(39):7466–9. 10.1002/anie.200802801 . 18729127

[36] Williamson DS, Borgognoni J, Clay A, Daniels Z, Dokurno P, Drysdale MJ, et al Novel adenosine-derived inhibitors of 70 kDa heat shock protein, discovered through structure-based design. J Med Chem. 2009;52(6):1510–3. 10.1021/jm801627a . 19256508

[37] Massey AJ, Williamson DS, Browne H, Murray JB, Dokurno P, Shaw T, et al A novel, small molecule inhibitor of Hsc70/Hsp70 potentiates Hsp90 inhibitor induced apoptosis in HCT116 colon carcinoma cells. Cancer Chemother Pharmacol. 2010;66(3):535–45. 10.1007/s00280-009-1194-3 . 20012863

[38] Ko SK, Kim J, Na DC, Park S, Park SH, Hyun JY, et al A small molecule inhibitor of ATPase activity of HSP70 induces apoptosis and has antitumor activities. Chem Biol. 2015;22(3):391–403. Epub 2015/03/17. 10.1016/j.chembiol.2015.02.004 . 25772468

[39] Leu JIJ, Pimkina J, Frank A, Murphy ME, George DL. A Small Molecule Inhibitor of Inducible Heat Shock Protein 70. Molecular Cell. 2009;36(1):15–27. 10.1016/j.molcel.2009.09.023 WOS:000271060500003. 19818706 PMC2771108

[40] Leu JI, Zhang P, Murphy ME, Marmorstein R, George DL. Structural basis for the inhibition of HSP70 and DnaK chaperones by small-molecule targeting of a C-terminal allosteric pocket. ACS Chem Biol. 2014;9(11):2508–16. Epub 2014/08/26. 10.1021/cb500236y 25148104 PMC4241170

[41] Wadhwa R, Sugihara T, Yoshida A, Nomura H, Reddel RR, Simpson R, et al Selective toxicity of MKT-077 to cancer cells is mediated by its binding to the hsp70 family protein mot-2 and reactivation of p53 function. Cancer Res. 2000;60(24):6818–21. . 11156371

[42] Rousaki A, Miyata Y, Jinwal UK, Dickey CA, Gestwicki JE, Zuiderweg ER. Allosteric drugs: the interaction of antitumor compound MKT-077 with human Hsp70 chaperones. J Mol Biol. 2011;411(3):614–32. 10.1016/j.jmb.2011.06.003 21708173 PMC3146629

[43] Li X, Srinivasan SR, Connarn J, Ahmad A, Young ZT, Kabza AM, et al Analogs of the Allosteric Heat Shock Protein 70 (Hsp70) Inhibitor, MKT-077, as Anti-Cancer Agents. ACS Med Chem Lett. 2013;4(11). Epub 2013/12/07. 10.1021/ml400204n 24312699 PMC3845967

[44] Rodina A, Patel PD, Kang Y, Patel Y, Baaklini I, Wong MJ, et al Identification of an allosteric pocket on human hsp70 reveals a mode of inhibition of this therapeutically important protein. Chem Biol. 2013;20(12):1469–80. 10.1016/j.chembiol.2013.10.008 24239008 PMC3985611

[45] Taldone T, Ochiana SO, Patel PD, Chiosis G. Selective targeting of the stress chaperome as a therapeutic strategy. Trends Pharmacol Sci. 2014;35(11):592–603. 10.1016/j.tips.2014.09.001 25262919 PMC4254259

[46] Lukacs GL, Verkman AS. CFTR: folding, misfolding and correcting the DeltaF508 conformational defect. Trends Mol Med. 2012;18(2):81–91. 10.1016/j.molmed.2011.10.003 22138491 PMC3643519

[47] Van Goor F, Straley KS, Cao D, Gonzalez J, Hadida S, Hazlewood A, et al Rescue of DeltaF508-CFTR trafficking and gating in human cystic fibrosis airway primary cultures by small molecules. Am J Physiol Lung Cell Mol Physiol. 2006;290(6):L1117–30. 10.1152/ajplung.00169.2005 . 16443646

[48] Van Goor F, Hadida S, Grootenhuis PD, Burton B, Stack JH, Straley KS, et al Correction of the F508del-CFTR protein processing defect in vitro by the investigational drug VX-809. Proc Natl Acad Sci U S A. 2011;108(46):18843–8. 10.1073/pnas.1105787108 21976485 PMC3219147

[49] Ren HY, Grove DE, De La Rosa O, Houck SA, Sopha P, Van Goor F, et al VX-809 corrects folding defects in cystic fibrosis transmembrane conductance regulator protein through action on membrane-spanning domain 1. Mol Biol Cell. 2013;24(19):3016–24. 10.1091/mbc.E13-05-0240 23924900 PMC3784376

[50] Van Goor F, Hadida S, Grootenhuis PD, Burton B, Cao D, Neuberger T, et al Rescue of CF airway epithelial cell function in vitro by a CFTR potentiator, VX-770. Proc Natl Acad Sci U S A. 2009;106(44):18825–30. 10.1073/pnas.0904709106 19846789 PMC2773991

[51] Wainwright CE, Elborn JS, Ramsey BW, Marigowda G, Huang X, Cipolli M, et al Lumacaftor-Ivacaftor in Patients with Cystic Fibrosis Homozygous for Phe508del CFTR. N Engl J Med. 2015;373(3):220–31. 10.1056/NEJMoa1409547 25981758 PMC4764353

[52] Bell SC, De Boeck K, Amaral MD. New pharmacological approaches for cystic fibrosis: promises, progress, pitfalls. Pharmacol Ther. 2015;145:19–34. 10.1016/j.pharmthera.2014.06.005 . 24932877

[53] Mall MA, Galietta LJ. Targeting ion channels in cystic fibrosis. J Cyst Fibros. 2015;14(5):561–70. 10.1016/j.jcf.2015.06.002 . 26115565

[54] Varga K, Goldstein RF, Jurkuvenaite A, Chen L, Matalon S, Sorscher EJ, et al Enhanced cell-surface stability of rescued DeltaF508 cystic fibrosis transmembrane conductance regulator (CFTR) by pharmacological chaperones. Biochem J. 2008;410(3):555–64. 10.1042/BJ20071420 18052931 PMC3939615

[55] Rab A, Bartoszewski R, Jurkuvenaite A, Wakefield J, Collawn JF, Bebok Z. Endoplasmic reticulum stress and the unfolded protein response regulate genomic cystic fibrosis transmembrane conductance regulator expression. Am J Physiol Cell Physiol. 2007;292(2):C756–66. 10.1152/ajpcell.00391.2006 . 16987996

[56] Perez-Sala D, Boya P, Ramos I, Herrera M, Stamatakis K. The C-terminal sequence of RhoB directs protein degradation through an endo-lysosomal pathway. PLoS One. 2009;4(12):e8117 10.1371/journal.pone.0008117 19956591 PMC2780327

[57] Farinha CM, Amaral MD. Most F508del-CFTR is targeted to degradation at an early folding checkpoint and independently of calnexin. Mol Cell Biol. 2005;25(12):5242–52. 10.1128/MCB.25.12.5242-5252.2005 15923638 PMC1140594

[58] Denning GM, Anderson MP, Amara JF, Marshall J, Smith AE, Welsh MJ. Processing of mutant cystic fibrosis transmembrane conductance regulator is temperature-sensitive. Nature. 1992;358(6389):761–4. 10.1038/358761a0 . 1380673

[59] Gee HY, Noh SH, Tang BL, Kim KH, Lee MG. Rescue of DeltaF508-CFTR trafficking via a GRASP-dependent unconventional secretion pathway. Cell. 2011;146(5):746–60. 10.1016/j.cell.2011.07.021 . 21884936

[60] Ma T, Thiagarajah JR, Yang H, Sonawane ND, Folli C, Galietta LJ, et al Thiazolidinone CFTR inhibitor identified by high-throughput screening blocks cholera toxin-induced intestinal fluid secretion. J Clin Invest. 2002;110(11):1651–8. 10.1172/JCI16112 12464670 PMC151633

[61] Taddei A, Folli C, Zegarra-Moran O, Fanen P, Verkman AS, Galietta LJ. Altered channel gating mechanism for CFTR inhibition by a high-affinity thiazolidinone blocker. FEBS Lett. 2004;558(1–3):52–6. 10.1016/S0014-5793(04)00011-0 . 14759515

[62] Matthes E, Goepp J, Carlile GW, Luo Y, Dejgaard K, Billet A, et al Low free drug concentration prevents inhibition of F508del CFTR functional expression by the potentiator VX-770 (ivacaftor). Br J Pharmacol. 2016;173(3):459–70. 10.1111/bph.13365 26492939 PMC4728415

[63] Hageman J, Kampinga HH. Computational analysis of the human HSPH/HSPA/DNAJ family and cloning of a human HSPH/HSPA/DNAJ expression library. Cell Stress Chaperones. 2009;14(1):1–21. 10.1007/s12192-008-0060-2 18686016 PMC2673897

[64] Finka A, Goloubinoff P. Proteomic data from human cell cultures refine mechanisms of chaperone-mediated protein homeostasis. Cell Stress Chaperones. 2013;18(5):591–605. 10.1007/s12192-013-0413-3 23430704 PMC3745260

[65] Zhang H, Amick J, Chakravarti R, Santarriaga S, Schlanger S, McGlone C, et al A bipartite interaction between Hsp70 and CHIP regulates ubiquitination of chaperoned client proteins. Structure. 2015;23(3):472–82. 10.1016/j.str.2015.01.003 25684577 PMC4351142

[66] Narayan V, Landre V, Ning J, Hernychova L, Muller P, Verma C, et al Protein-Protein Interactions Modulate the Docking-Dependent E3-Ubiquitin Ligase Activity of Carboxy-Terminus of Hsc70-Interacting Protein (CHIP). Mol Cell Proteomics. 2015;14(11):2973–87. 10.1074/mcp.M115.051169 26330542 PMC4638040

[67] Bhangoo MK, Tzankov S, Fan AC, Dejgaard K, Thomas DY, Young JC. Multiple 40-kDa heat-shock protein chaperones function in Tom70-dependent mitochondrial import. Mol Biol Cell. 2007;18(9):3414–28. 10.1091/mbc.E07-01-0088 17596514 PMC1951752

[68] Tzankov S, Wong MJ, Shi K, Nassif C, Young JC. Functional divergence between co-chaperones of Hsc70. J Biol Chem. 2008;283(40):27100–9. 10.1074/jbc.M803923200 . 18684711 PMC5026489

[69] Orthwein A, Zahn A, Methot SP, Godin D, Conticello SG, Terada K, et al Optimal functional levels of activation-induced deaminase specifically require the Hsp40 DnaJa1. EMBO J. 2012;31(3):679–91. 10.1038/emboj.2011.417 22085931 PMC3273393

[70] Terada K, Yomogida K, Imai T, Kiyonari H, Takeda N, Kadomatsu T, et al A type I DnaJ homolog, DjA1, regulates androgen receptor signaling and spermatogenesis. EMBO J. 2005;24(3):611–22. 10.1038/sj.emboj.7600549 15660130 PMC548655

[71] Rosales-Hernandez A, Beck KE, Zhao X, Braun AP, Braun JE. RDJ2 (DNAJA2) chaperones neural G protein signaling pathways. Cell Stress Chaperones. 2009;14(1):71–82. 10.1007/s12192-008-0056-y 18595009 PMC2673899

