# Supplementary figures and images for: Hsp70 and DNAJA2 limit CFTR levels through degradation
Source: PLoS One. 2019 Aug 13;14(8):e0220984. doi: 10.1371/journal.pone.0220984 (PMC6692068; doi:10.1371/journal.pone.0220984)

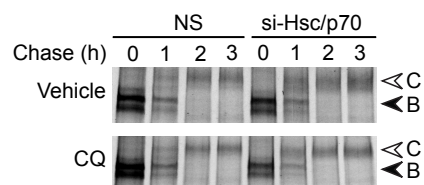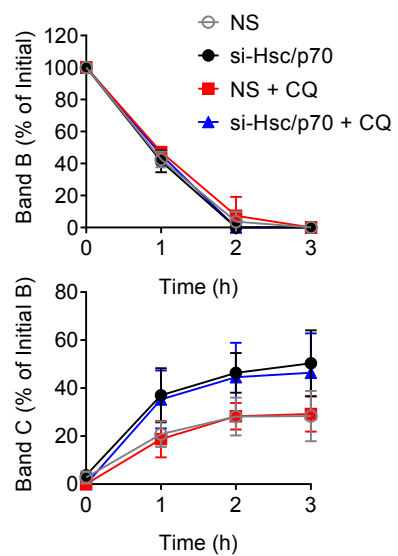

Supplement: S1 Fig — CFTR-3HA was stably expressed in HeLa cells transfected with siRNA against Hsc70 and Hsp70 (si-Hsc/p70) or non-silencing (NS) siRNA. Pulse-chase autoradiograph of CFTR-3HA is shown, with quantitations of bands B and C relative to initial amounts of band B, n = 3. Error bars show standard deviation from the mean. (PDF) [file pone.0220984.s001.pdf]

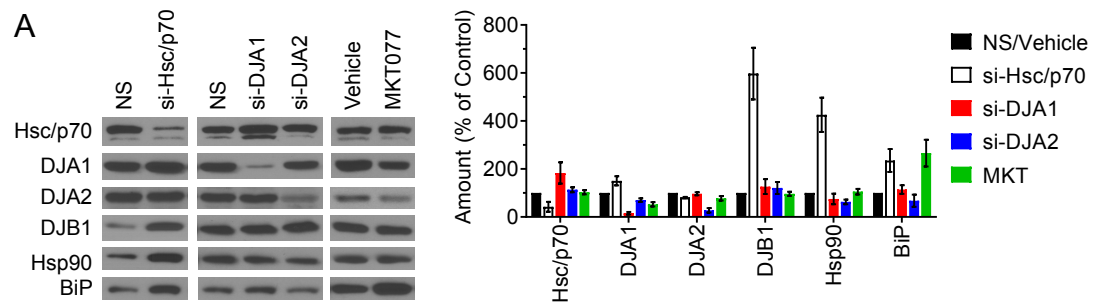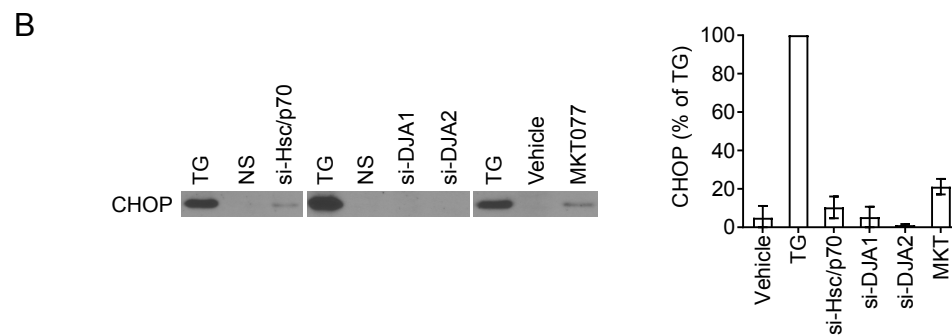

Supplement: S2 Fig — (A) Induction of chaperones. HeLa cells stably expressing CFTR-3HA were transfected with siRNA against Hsc70 and Hsp70 (si-Hsc/p70), or DJA1 (si-DJA1), or DJA2 (si-DJA2), or non-silencing (NS) siRNA, or 10 μM MKT077 or vehicle control for 24 h. The indicated chaperones were detected by immunoblot, and quantitations relative to non-silencing or vehicle controls are shown; si-Hsc/p70 n = 3; si-DJA1, si-DJA2 and MKT077, n = 4. (B) Induction of CHOP. Cells were transfected with siRNA as above, or treated with 0.1 μM thapsigargin (TG), or 10 μM MKT077, or vehicle control for 24 h. CHOP was detected by immunoblot, and quantitations relative to maximum induction by thapsigargin are shown; si-Hsc/p70, si-DJA1 and si-DJA2, n = 2; MKT077, n = 4. Error bars show standard deviation from the mean. (PDF) [file pone.0220984.s002.pdf]

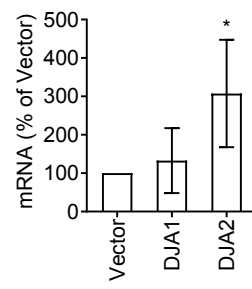

Supplement: S3 Fig — HEK293 cells were transfected with CFTR-3HA and myc-DJA1, myc-DJA2 or vector control. Total mRNA was extracted and reverse-transcribed, amounts of CFTR cDNA were determined by quantitative PCR normalized to actin cDNA. Quantitations relative to amounts in vector controls are shown, n = 5. Error bars show standard deviation from the mean, * p<0.05. (PDF) [file pone.0220984.s003.pdf]

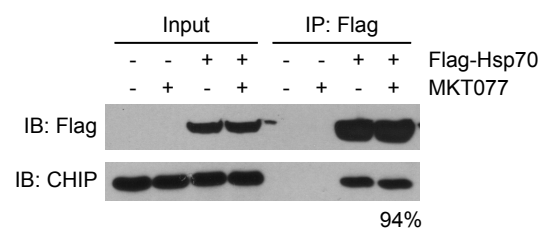

Supplement: S4 Fig — HeLa cells stably expressing CFTR-3HA were transfected with Flag-Hsp70 or empty vector, and treated with MKT077 or vehicle control. Flag-Hsp70 was immunoprecipitated (IP) and bound CHIP detected by immunoblot (IB). Quantified CHIP was normalized to total expression and amount of Hsp70 in the IP, and shown as a percentage of the IP with Flag-Hsp70 and vehicle control. (PDF) [file pone.0220984.s004.pdf]

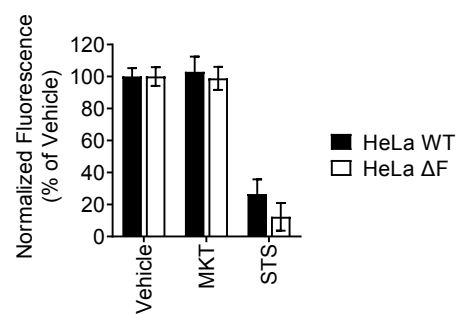

Supplement: S5 Fig — HeLa cells stably expressing CFTR-3HA or ΔF508-CFTR-3HA were treated with 10 μM MKT077, or 1 μM staurosporine (STS), or vehicle control for 24 hours. Viability was determined by fluorescence of Alamar blue reagent, normalized to total protein amounts. Quantitations relative to vehicle controls are shown, n = 4. Error bars show standard deviation from the mean. (PDF) [file pone.0220984.s005.pdf]

## Endoplasmic Reticulum

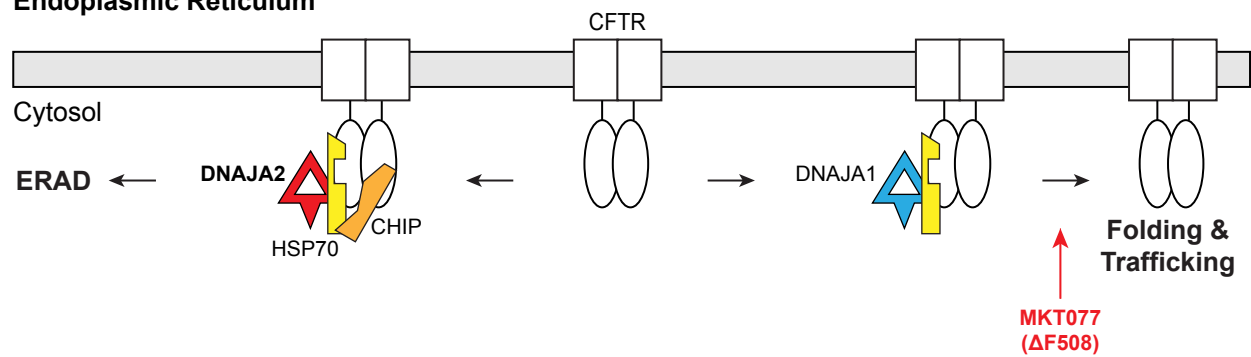

## Plasma Membrane

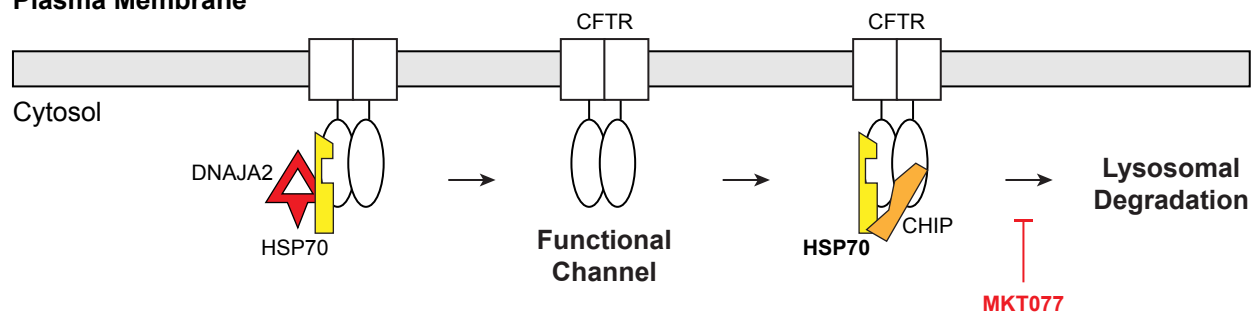

Supplement: S6 Fig — At the ER, DNAJA1 assists the folding of immature CFTR. Excess DNAJA2 and Hsp70 promote ERAD of CFTR through the E3 ligase CHIP. At the PM, Hsp70 promotes the degradation of mature CFTR in lysosomes, also through CHIP. Hsp70 inhibitor MKT077 increases mature CFTR by inhibiting its degradation. MKT077 also increases mature ΔF508-CFTR by allowing its slow maturation and accumulation. (PDF) [file pone.0220984.s006.pdf]
